# Fast, efficient, narrowband room-temperature phosphorescence from metal-free 1,2-diketones: rational design and the mechanism†

**DOI:** 10.1039/d4sc02841d

**Published:** 2024-06-03

**Authors:** Yosuke Tani, Kiyoshi Miyata, Erika Ou, Yuya Oshima, Mao Komura, Morihisa Terasaki, Shuji Kimura, Takumi Ehara, Koki Kubo, Ken Onda, Takuji Ogawa

**Affiliations:** a Department of Chemistry, Graduate School of Science, Osaka University Machikaneyama 1-1 Toyonaka Osaka 560-0043 Japan y-tani@chem.sci.osaka-u.ac.jp; b Innovative Catalysis Science Division, Institute for Open and Transdisciplinary Research Initiatives (ICS-OTRI), Osaka University Suita Osaka 560-8531 Japan; c Department of Chemistry, Faculty of Science, Kyushu University 744 Motooka Nishi Fukuoka 819-0395 Japan kmiyata@chem.kyushu-univ.jp

## Abstract

We report metal-free organic 1,2-diketones that exhibit fast and highly efficient room-temperature phosphorescence (RTP) with high colour purity under various conditions, including solutions. RTP quantum yields reached 38.2% in solution under Ar, 54% in a polymer matrix in air, and 50% in crystalline solids in air. Moreover, the narrowband RTP consistently dominated the steady-state emission, regardless of the molecular environment. Detailed mechanistic studies using ultrafast spectroscopy, single-crystal X-ray structure analysis, and theoretical calculations revealed picosecond intersystem crossing (ISC) followed by RTP from a planar conformation. Notably, the phosphorescence rate constant *k*_p_ was unambiguously established as ∼5000 s^−1^, which is comparable to that of platinum porphyrins (representative heavy-metal phosphor). This inherently large *k*_p_ enabled the high-efficiency RTP across diverse molecular environments, thus complementing the streamlined persistent RTP approach. The mechanism behind the photofunction has been elucidated as follows: (1) the large *k*_p_ is due to efficient intensity borrowing of the T_1_ state from the bright S_3_ state, (2) the rapid ISC occurs from the S_1_ to the T_3_ state because these states are nearly isoenergetic and have a considerable spin–orbit coupling, and (3) the narrowband emission results from the minimal geometry change between the T_1_ and S_0_ states. Such mechanistic understanding based on molecular orbitals, as well as the structure-RTP property relationship study, highlighted design principles embodied by the diketone planar conformer. The fast RTP strategy enables development of organic phosphors with emissions independent of environmental conditions, thereby offering alternatives to precious-metal based phosphors.

## Abstract 2

Fast room-temperature phosphorescence with high quantum yields up to 38% in solution from metal-free organic 1,2-diketones is reported, along with the mechanism and molecular design principles governing the fast phosphorescence.

## Graphical abstract

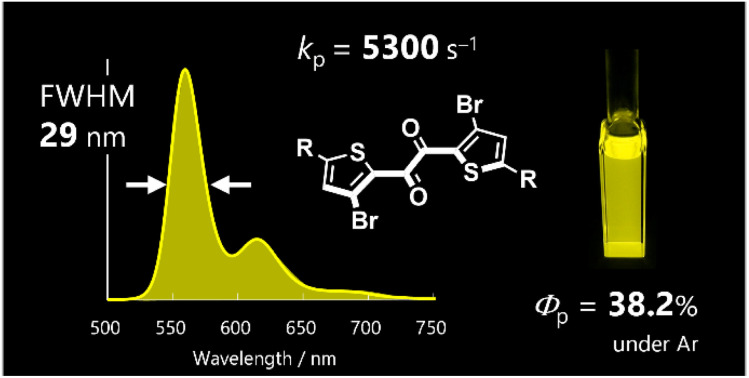

## Introduction

Room-temperature phosphorescence (RTP) from metal-free organic molecules has been an area of intense research.^1^ Although classical RTP materials have been used for diverse applications, including organic light-emitting diodes (OLEDs) and bioimaging, they are mainly precious-metal complexes of Ir or Pt.^2^ Therefore, cost-effective, less-toxic, and sustainable metal-free alternatives are needed. However, intrinsic molecular RTP of metal-free phosphors has not rivalled that of metal complexes.

**Fig. 1:**
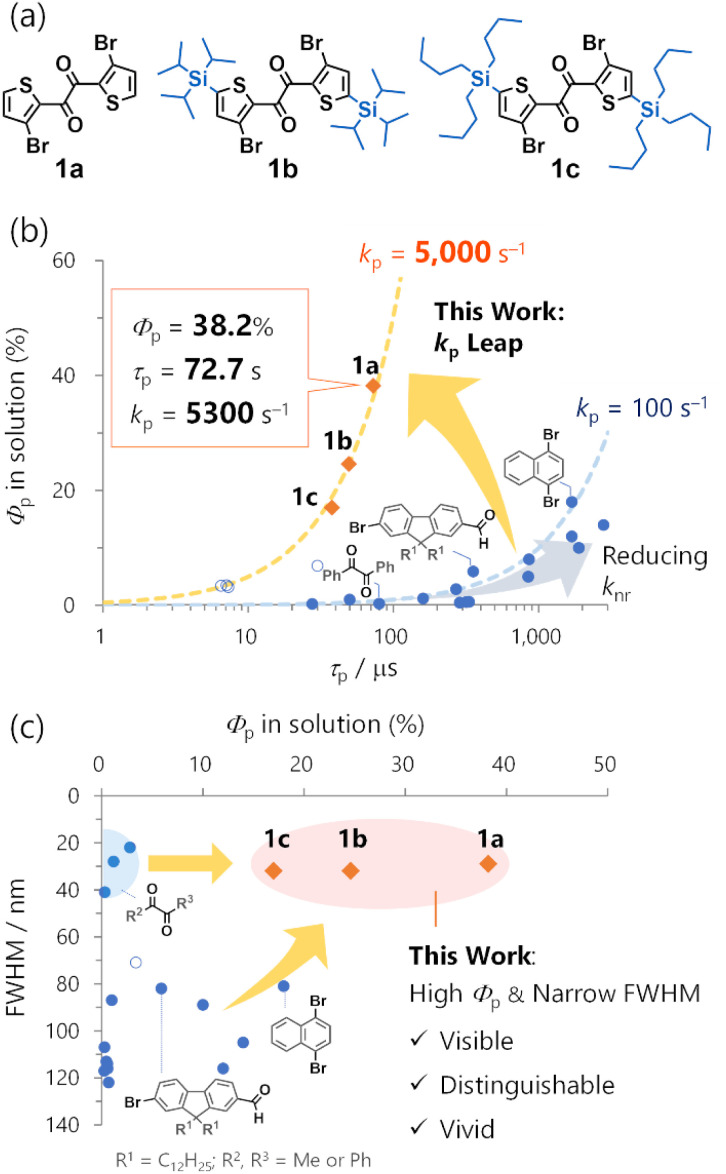
Comparison of representative metal-free organic phosphors (blue filled circles), thiocarbonyls (blue open circles), and 1 (orange diamonds) in solution. (a) Molecular structures of 1. (b) Room-temperature phosphorescence quantum yields (*Φ*_p_) *vs.* lifetimes (*τ*_p_); (c) full-width-at-half-maxima (FWHM) *vs. Φ*_p_. Blue and orange broken lines in (b) represent *k*_p_ = 100 and 5000 s^−1^, respectively, assuming unity intersystem crossing quantum yields.

Organic RTP must overcome several challenges stemming from the absence of heavy-metal atoms. The most severe is the inherently small phosphorescence transition probability (*i.e.*, rate constant *k*_p_), causing poor RTP quantum yields (*Φ*_p_). *k*_p_ can be 10^4^–10^5^ s^−1^ for heavy-metal complexes,^3^ and only ∼10 s^−1^ or less for conventional organic compounds.^1*a*^ Introducing heavy atoms, such as bromine, iodine, selenium, and tellurium, in conjunction with carbonyl functionalities, is a classical approach for enhancing *k*_p_.^4^ Despite its long history, however, an organic *k*_p_ over ∼100 s^−1^ and/or a *Φ*_p_ over 1% in solutions are rarely observed (Fig. 1b, blue filled circles; Fig. S1 and Table S1†).^5^ The use of thiocarbonyls could provide a large *k*_p_ in some cases (Fig. 1b, blue open circles).^6^ However, they are associated with high (photo)chemical reactivities and instability,^7^ thereby limiting their high *Φ*_p_ in perfluoroalkane solvents.^6*b*^ The only practical way to improve *Φ*_p_ has been to reduce the nonradiative decay rate constant (*k*_nr_). This approach has provided solid-state phosphors with RTP lasting for subseconds after excitation cessation (persistent RTP or afterglow).^8^ However, it inevitably restricts the organic phosphors to rigid media, such as crystals,^9^ or tailored host–guest systems with strong intermolecular interactions.^10^

A promising direction for environmentally independent organic RTP could be the significant increase in *k*_p_s. Complementary to the persistent RTP, fast RTP could have significant potential use in solutions or any non-rigid molecular environment, including biological conditions.^11^ Moreover, because fast RTP can avoid efficiency loss caused by the accumulation of the triplet excitons, it also could be used in optoelectronics such as OLEDs.^12^ However, the quest for fast RTP is facing difficulty so far. In 2022, a selenium-containing molecule (phenoxaselenine) was designed as a candidate for fast RTP; however, its RTP was weak even in a polymer matrix, and only the photoluminescence spectrum and intrinsic phosphorescence rate (*ca.* 4000 s^−1^) at 78 K were reported.^13^ Quite recently, organic ionic crystals were reported to exhibit fast RTP with impressive *k*_p_ values up to ∼10^5^ s^−1^.^14^ The authors ascribed the large *k*_p_ to the external heavy atom effect of two appropriately arranged iodide counter anions. Nonetheless, such effects relied on the crystal packing, and no RTP was observed in solutions. Thus, molecular design for a large *k*_p_ that enables RTP in solution is needed.

Low colour purity is another organic RTP challenge. Considering the luminescence of the same quantum efficiency, high-colour-purity emission has a sharp and intense spectral peak that is visible, distinguishable, and vivid. These are essential features for OLED displays and bioimaging.^15^ However, organic RTP is usually broad with 80–120 nm full-width at half-maxima (FWHMs) (Fig. 1b, Table S1†). Moreover, because of an inefficient intersystem crossing (ISC) from singlet to triplet states, organic phosphors often exhibit both RTP and fluorescence, further impairing colour purity. As a long-known exception, 1,2-diketones, such as biacetyl and benzil, exhibit narrowband RTP that consists of one main peak with a small FWHM value accompanied by weak vibronic bands.^16^ However, the RTP is feeble in solution. Thus, simultaneously achieving a high *Φ*_p_ and colour purity in organic RTP remains a tremendous challenge.

Using heteroaromatic 1,2-diketones, we previously developed a series of solid-state mechanoresponsive RTP materials,^17^ solvent-free liquid RTP materials,^18^ and photoresponsive RTP crystals (Fig. S3†).^19^ They exhibited RTP in non-rigid molecular environments, such as amorphous solids or solvent-free liquids, wherein conventional metal-free organic compounds rarely show RTP. Thus, our previous results not only demonstrated unique advantages of the 1,2-diketone-based materials in applications, but also implied large *k*_p_s. However, the fundamental molecular RTP properties remain elusive due to the complexity of the condensed materials. While we have briefly reported some solution-phase RTP properties of thienyl diketones 1a and 1b,^17*a*^ further investigations were necessary to evaluate *k*_p_ quantitatively (Fig. 1a and S2†). Typically, *k*_p_ is derived from experimental *Φ*_p_, ISC quantum yield *ϕ*_ISC_, and RTP lifetime *τ*_p_, expressed as *k*_p_ = *Φ*_p_/(*ϕ*_ISC_ × *τ*_p_). However, *ϕ*_ISC_ was not evaluated in our prior research as we did not focus on *k*_p_. In addition, while we determined *Φ*_p_ and *τ*_p_ of 1b, the previous protocol did not assure consistent degassing for those measurements (*i.e.*, the extent of oxygen quenching of RTP would be different; Fig. S2†). Consequently, these values cannot be employed to establish *k*_p_. Therefore, the mechanism governing the expected fast RTP remained unexplored, not to mention the absence of the molecular design principle for substantial *k*_p_.

Here, we disclose the inherent molecular RTP properties of the thienyl diketone derivatives 1, exhibiting high-efficiency narrowband RTP based on an exceptionally large *k*_p_s of ∼5000 s^−1^ (Fig. 1). In particular, 1a exhibited 38.2% *Φ*_p_ in cyclohexane. To our knowledge, this is the highest efficiency for metal-free organic molecules in common solvents. Relative to benzil, the *k*_p_ was increased by a factor of ∼100 from 39 s^−1^ to 5300 s^−1^, enabling a 100-time increase in *Φ*_p_ from 0.31% to 38% (Fig. 1a).^16*c*^ Moreover, high *Φ*_p_ with a narrow ∼30 nm FWHM was observed in solution (Fig. 1b). The high efficiency and colour purity were also observed in various conventional polymer matrices and crystalline solids, indicating that the RTP was an inherent molecular feature. The RTP mechanism and origin were revealed through single-crystal X-ray structure analysis, time-resolved photoluminescence (TRPL) spectroscopy, transient absorption (TA) spectroscopy, time-resolved infrared (TRIR) spectroscopy, and quantum-chemical calculations. Furthermore, structure-RTP property relationship study considering molecular- and natural transition orbitals provided design principles for fast organic RTP. Our work demonstrates the potential of metal-free organic molecular materials to exhibit fast RTP comparable to precious-metal complexes. Complementary to persistent RTP, organic fast RTP is promising not only for application in solution but also in functional condensed materials as we have witnessed in our previous studies (Fig. S3†).

## Results and discussion

### RTP and related rate constants of 1a

**Fig. 2:**
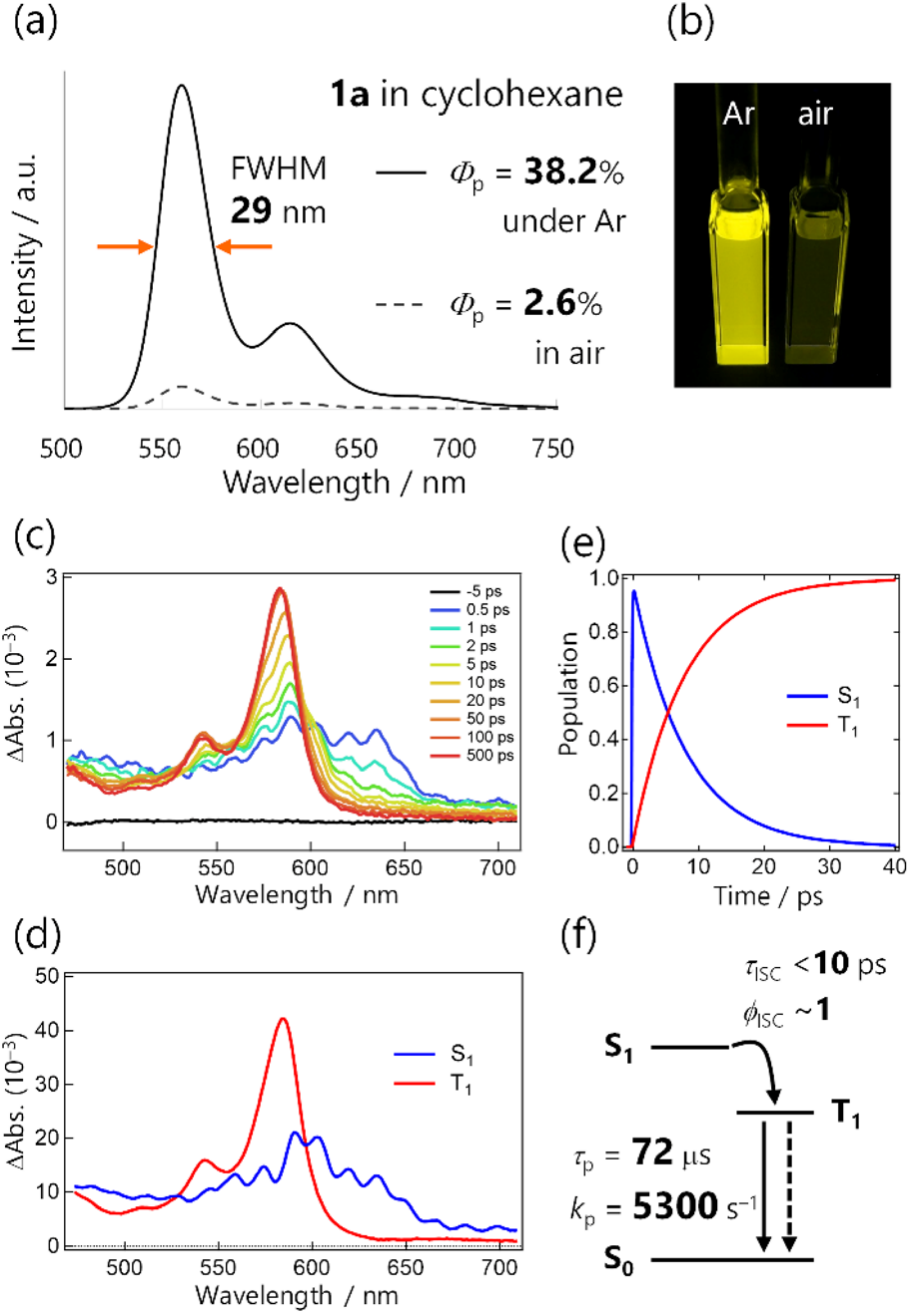
(a) Steady-state photoluminescence spectra of 1a in cyclohexane (4.4 × 10^−6^ M, excited at 368 nm). (b) Photograph of solutions under 365 nm excitation. (c) fs transient absorption spectra of 1a in cyclohexane excited at 355 nm. (d and e) Selected results from the global analysis based on a sequential model; (d) evolution-associated spectra and (e) corresponding concentration kinetics. Coherent artefact signals are omitted for clarity. (f) Schematic summary of the excited-state dynamics in 1a.

We first examined the RTP properties in solution for the diketone 1a, which exhibits strong yellow emission (Fig. 2a and b). The steady-state photoluminescence spectrum of 1a in cyclohexane had a single, sharp emission peak at 560 nm accompanied by weak vibronic bands at 615 and ∼680 nm.^20^ The total photoluminescence quantum yield *Φ*_PL_ was 38.2% under Ar and the lifetime was 72.7 μs, as determined with a protocol that assures consistent degassing for both measurements (Scheme S2†). TRPL spectra of 1a revealed that the emission emerged just after photoexcitation (within a <100 ps instrumental response function), and exhibited a long lifetime (Fig. S11†). Hence, the emission was phosphorescence, as reported previously for thienyl diketone analogs,^17^ and ISC occurred over a timescale less than the instrumental response.

To quantify the rapid ISC, we conducted femtosecond TA spectrum (fsTAS) measurement with ∼100 fs time resolution (Fig. 2c). The broad transient absorption over 450–650 nm changed to sharp spectra with a peak at 580 nm in a ∼10 ps timescale. We globally analysed the TAS by using a sequential model assuming two excited species, resulting in successful fitting with the time constant of the transition estimated to be 7.9 ps (Fig. 2d and e and S14†). We also conducted TAS measurements over nanosecond timescales (Fig. S13†), and observed that the TAS shape was unchanged up to the microsecond timescale. The long-lived spectral component was the lowest triplet excited state (T_1_ state), populated *via* ISC with a time constant of <10 ps for 1a in solution (Fig. 2f). Because the ISC outcompeted other relaxation processes, we can reasonably assume that the ISC quantum yield (*ϕ*_ISC_) was unity.

The total photoluminescence quantum yield can be expressed as *Φ*_PL_ = *Φ*_f_ + *Φ*_p_, where *Φ*_f_ is the fluorescence quantum yield. Given that *ϕ*_ISC_ ∼ 1, phosphorescence dominates the total emission, and *Φ*_p_ ∼ *Φ*_PL_ = 0.38. To the best of our knowledge,^21–23^ this is a record-breaking RTP efficiency for metal-free organic molecules in common solvents. In addition, with *ϕ*_ISC_ ∼ 1 confirmed, *Φ*_p_ = *ϕ*_ISC_*k*_p_*τ*_p_ ∼ *k*_p_*τ*_p_. For *Φ*_p_ = 0.38 and *τ*_p_ = 72.7 μs, *k*_p_ was derived to be 5300 s^−1^, which greatly exceeded those of previously reported organic phosphors, except for thiocarbonyl compounds (Fig. 1a and Table S1†). Because of this exceptionally high *k*_p_, the RTP of 1a in solution was visible even in air (*Φ*_p_ = 2.6%, Fig. 2a and b, S10, Table S2, and ESI Movie 1†). We note that the estimation of *k*_p_ in the literature often assumed *ϕ*_ISC_ = 1 and *Φ*_p_ ∼ *Φ*_PL_ without evaluating the time constant of ISC, and employed *Φ*_p_ and *τ*_p_ that were determined without assuring consistent degassing. This would be practical for qualitative purposes, but may cause significant errors in the *k*_p_ value. Experimental evaluation of *ϕ*_ISC_ and assuring consistent degassing are essential for the quantitative evaluation of *k*_p_.

**Fig. 3:**
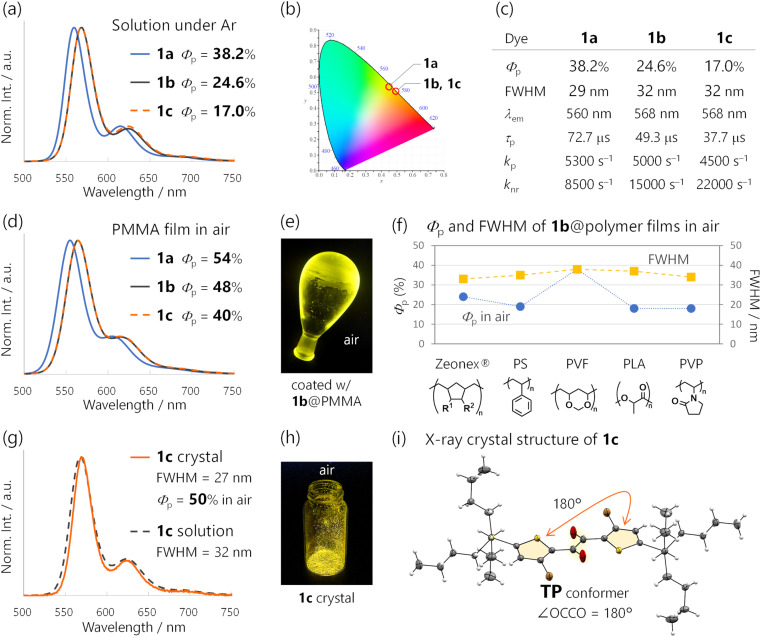
(a) Steady-state photoluminescence (PL) spectra of 1a–1c in cyclohexane (10–4.4 × 10^−6^ M, excited at 368 nm) and (b) CIE1931 chromaticity diagram for their PL in cyclohexane. (c) Photophysical properties of 1a–1c in cyclohexane under Ar (10–4.4 × 10^−6^ M, excited at 368 nm). *Φ*_p_, RTP quantum yields; FWHM, full-width-at-half-maxima; *λ*_em_, emission maxima; *τ*_p_, lifetimes; *k*_p_ and *k*_nr_, phosphorescence and nonradiative rate constants. (d and g) Steady-state PL spectra of (d) 1a–1c@PMMA (5 wt%, excited at 355–368 nm) and (g) a 1c crystal in cyclohexane (1.0 × 10^−5^ M, excited at 368 nm). (e and h) Photographs of (e) 1b@PMMA and (h) a 1c crystal under 365 nm excitation. (f) *Φ*_p_ in air and the FWHM of 1b-doped polymer films (5 wt%, excited at 350–375 nm). (i) ORTEP drawing of the crystal structure of 1c. Thermal ellipsoids are set at the 50% probability level.

The emission colour purity is also outstanding. Fluorescence was not discernible in the steady-state photoluminescence, and the FWHM of the RTP was only 29 nm for 1a (Fig. 2a and S10†). The RTP contained two other vibronic bands at around 615 and 680 nm; nonetheless, the first band at 560 nm covered 71% of the whole spectral area (Table S3†). This is distinct from other organic phosphors, which have broad and structure-less spectra with a 80–120 nm FWHM (Fig. 1c and Table S1†). Although there is a room for further improvement, the narrowband RTP with weak vibronic bands and the fluorescence-free nature represented a high colour purity emission; its coordinates (0.45, 0.54) are located almost at the edge of the chromaticity diagram of Commission internationale de l'éclairage (CIE) 1931 (Fig. 3b).

### Substituent effect

Next, we investigated the effects of trialkylsilyl substituents on the RTP properties in solution. Trialkylsilyl substituents are known to perturb π-electronic systems through σ–π and/or σ*–π* conjugation (hyperconjugation). While these substituents sometimes improve the fluorescence efficiency, their effects on RTP properties have gained less attention.^9*d*,24^

We introduced triisopropylsilyl (1b) and tributylsilyl groups (1c) onto the thienyl diketone core (Fig. 1a) and examined their properties in cyclohexane solution. The silylated derivatives 1b and 1c provided identical emission spectra with the maxima slightly red-shifted from 560 nm to 568 nm compared to those of 1a (Fig. 3a and S10†). The steady-state emissions were fluorescence-free and maintained a small FWHM (32 nm). This narrowband yellow emission had a CIE 1931 coordinate of (0.49, 051) (Fig. 3b).^2*c*^ We also conducted fsTAS measurement for 1b (Fig. S15†) to analyse ISC; the main feature of the TAS was largely the same as that for 1a and the ISC time constants were 0.63 and 2.3 ps. The time constant is even faster than that of 1a and we can assume unity *ϕ*_ISC_ for the silylated diketones. *Φ*_p_ for 1b and 1c was less than that for 1a, but still 24.6 and 17%, respectively (Fig. 3c). The decrease in *Φ*_p_ could be attributed to the doubled *k*_nr_ values, from 8500 s^−1^ for 1a to 15000 s^−1^ and 22000 s^−1^ for 1b and 1c, respectively. This was most likely because of the nonradiative decay accelerated by molecular motion involving the silyl groups. In contrast, *k*_p_ values were only slightly changed, 5000 and 4500 s^−1^ for 1b and 1c. These results indicated that the large *k*_p_ of 1 basically originated from the thienyl diketone core.

### RTP of 1-doped polymer films in air

The huge *k*_p_ of 1 enabled efficient RTP in air in the amorphous state using various conventional polymers as matrices (Fig. 3d–f and S20–S24 and Table S4†). The best *Φ*_p_ of 54% was achieved at room temperature in air for a poly(methyl methacrylate) (PMMA) film doped with 5-wt% 1a (Fig. 3d). The PMMA films doped with silylated diketones 1b and 1c also provided narrowband PL spectra similar to that of 1a and exhibited a high *Φ*_p_ of 48 and 40%, respectively (Fig. 3d and e). These were among the highest reported for polymer-based RTP systems doped with metal-free organic dyes.^25^ The reported systems with insufficient *k*_p_ required a tailor-made approach that involves specific interactions between the polymers and dopants. Hydrogen bonding in poly(vinyl alcohol) was reported to be effective and widely used,^10*c*–*e*^ but these interactions or polymers are difficult to use under highly humid conditions or in water. Because PMMA is resistant to water, the RTP properties of a 1c-doped PMMA film were not spoiled by water, exhibiting the same PL spectrum with a *Φ*_p_ of 35% even when submerged in water (Fig. S22†).^26^

Moreover, phosphorescent films with *Φ*_p_ values of 38–18% were obtained by doping 1b into other five conventional polymers (Fig. 3f and S23, S24 and Table S4†). The emission maxima and FWHMs were almost unchanged, with colour purities independent of the polymers. Thus, RTP properties of the films were derived from inherent characteristics of 1.

We would like to emphasize that achieving efficient organic RTP in solution and in polymer matrices is challenging because nonradiative decay is not suppressed under these conditions. In such cases, the fast RTP (large *k*_p_) has a distinct advantage over persistent RTP with small *k*_p_. The same can be said for the RTP in non-crystalline aggregate states, such as the amorphous solid state and solvent-free liquid state. It becomes clear that our previous studies of mechanoresponsive RTP materials,^17^ solvent-free liquid RTP materials,^18^ and photoresponsive RTP crystals^19^ (Fig. S3†) demonstrated the usefulness and applications of fast RTP.

### Conformation of RTP-emitting species

Considering that 1 has three successive single bonds in the diketone core, identifying the RTP-emitting conformation was important.^16*b*–*d*,27^ In our previous study, two distinct conformers of thienyl diketones were identified by single-crystal X-ray structure analysis.^17*a*^ The 1a crystal exhibited a *trans*-planar (TP) conformation with respect to the dicarbonyl moiety, while the 1b crystal exhibited a skew conformation (Fig. S33†). Thus, the conformation in crystals varied based on the substituents due to subtle differences in the intermolecular interactions. Unfortunately, the 1a crystal was nonemissive, likely due to intermolecular electronic interactions. 1a exhibited one-dimensional columnar π-stacking with a 3.481 Å interplanar distance, and the absorption of the crystal had a long tail until over 700 nm (Fig. S34†). On the other hand, the 1b crystal emitted green RTP, which was different from the solution RTP. Therefore, further study was required to nail down the conformation of the yellow RTP-emitting species.

In the present work, we found that crystals of 1c exhibited yellow RTP with a 50% *Φ*_p_ in air (Fig. 3g and h). The RTP decayed as a single exponential with a *τ*_p_ of 79 μs, and *k*_p_ was 6000 s^−1^ (Fig. S25†). Most importantly, the PL spectrum was almost identical to that of 1c in solution, indicating emission from the same conformer (Fig. 3g). Single-crystal X-ray structure analysis revealed the TP conformation of 1c, with the thienyl diketone core spatially separated from neighbouring molecules by tributylsilyl substituents (Fig. 3i and S32†; no π-stacking, and the interplanar distance was 4.45 Å). Thus, the RTP from the 1c crystal, as well as 1 in solution, was unambiguously assigned to the monomer emission from its TP conformer. Notably, 1c represents a rare example of metal-free organic molecules exhibiting remarkable *Φ*_p_ in solution, in a polymer matrix, and in a crystal, with almost identical PL spectra.

To experimentally elucidate conformation dynamics in the excited state, TRIR spectroscopy was performed. Although we could not perform the TRIR measurement on 1a because of poor solubility, we managed to conduct the TRIR measurement on 1b in cyclohexane using a 267 nm wavelength pump light (Fig. S16†). The spectra converged to sharp spectra over 0.74 ps and 2.67 ps timescales, consistent with the time constants extracted from fsTAS of 1b (0.63 and 2.3 ps, Fig. S15†). Note that these time constants were comparable to those observed for 1a in the fsTAS measurement. This indicated that the excited-state dynamics were primarily affected by the thienyl diketone core, while the silyl groups had minor effects. The two timescales were attributed to the conformation change and ISC. More importantly, the converged TRIR spectra were consistent with simulated spectra, assuming a TP conformation in the T_1_ state (Fig. S17†). Thus, both the conformation changes and the ISC were ultrafast, and the RTP is confirmed to originate from the TP conformer.

### Origin of the rapid ISC

**Fig. 4:**
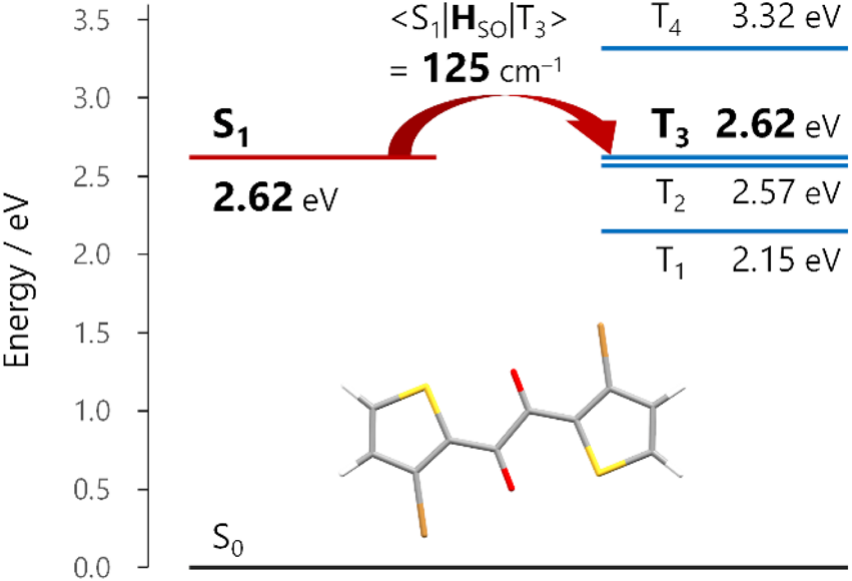
Energy diagram of 1a in the S_1_-optimized *trans*-planar (TP) geometry. Energies and the spin–orbit coupling matrix element between the S_1_ and T_3_ states 〈S_1_|**H**_SO_|T_3_〉 were calculated at the TDA/uCAM-B3LYP-D3/6-311G(d) level of theory.

Given that the ISC occurs at the TP conformation, we investigated the molecular origin of the rapid ISC by time-dependent density functional theory (TDDFT) calculations (Fig. 4 and S26†). In principle, ISC is fast when the energy gap is small and the spin–orbit coupling (SOC) matrix element is large.^28^ In the S_1_-optimized TP geometry, the S_1_ state was almost isoenergetic to the T_3_ state (energy gap <0.01 eV for 1a and 0.04 eV for 1b at the TDA/uCAM-B3LYP-D3/6-311G(d) level of theory).^29^ Moreover, the S_1_–T_3_ SOC matrix elements 〈S_1_|**H**_SO_|T_3_〉 for 1a and 1b were 125 and 112 cm^−1^, respectively. The substantial SOC matrix elements were consistent with El Sayed's rule; the electronic configurations of the S_1_ and T_3_ states were (n,π*) and (π,π*), respectively (Fig. S26†). These results strongly supported the ultrafast ISC, which occurred dominantly from the S_1_ to T_3_ states, followed by internal conversion to the T_1_ state.

### Origin of the substantial *k*_p_ value

Based on concrete basis that the RTP comes from the TP conformer, we then investigated the origin of the outstanding *k*_p_. Theoretically, it was roughly based on intensity borrowing from the *n*th excited singlet states (S_*n*_), as given by:^28*a*^1
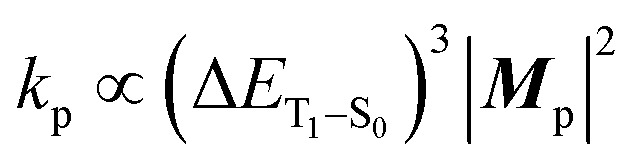
2
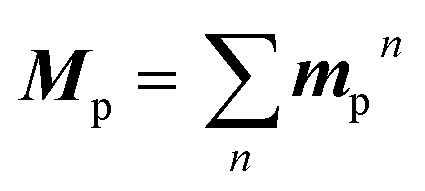
3
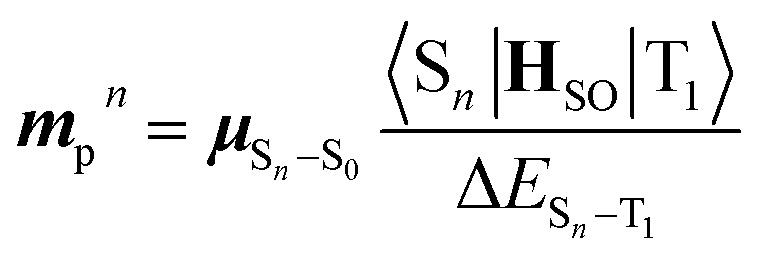
where ***M***_p_ and ***μ***_S_*n*_–S_0__ are the T_1_–S_0_ and S_*n*_–S_0_ transition dipole moments at the T_1_-geometry, respectively; ***m***_p_^*n*^ contributed to ***M***_p_ by borrowing intensity from the S_*n*_ state; 〈S_*n*_|**H**_SO_|T_1_〉 is the SOC matrix element between the S_*n*_ and T_1_ states. Eqn (1)–(3) provide two insights: (i) ***m***_p_^*n*^, and hence *k*_p_, increase when S_*n*_ states of a large (spin-allowed) ***μ***_S_*n*_–S_0__ couple with the T_1_ state; and (ii) the coupling increases when SOC with energetically close S_*n*_ states is effective; 〈S_*n*_|**H**_SO_|T_1_〉/Δ*E*_S_*n*_–T_1__ can be regarded as the mixing coefficient. It should be emphasized that a large SOC matrix element is insufficient because matching of the three factors in eqn (3)*via* the same S_*n*_ states is the requisite for a significant *k*_p_.

**Fig. 5:**
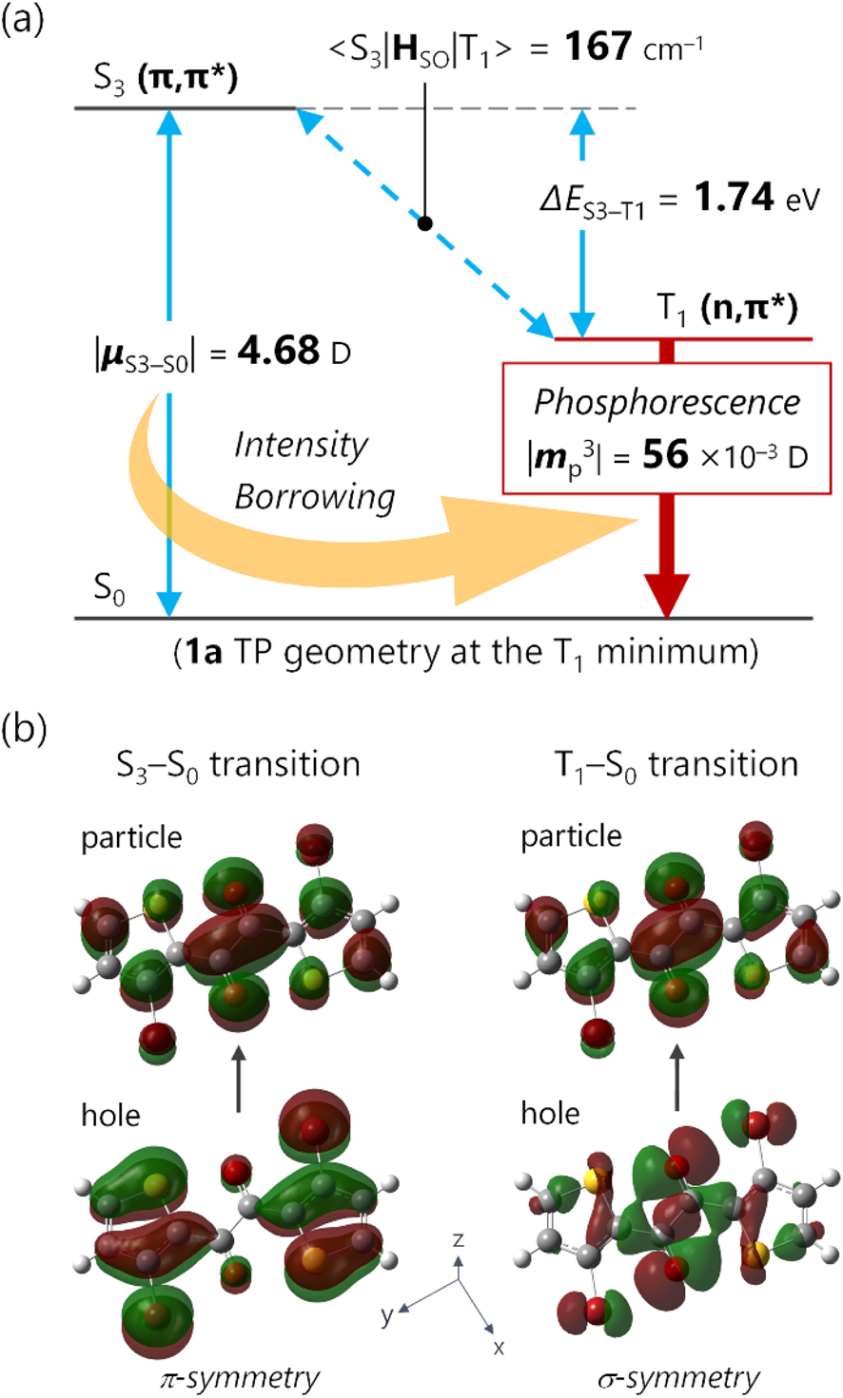
(a) Energy diagram of 1a in the T_1_-optimized *trans*-planar (TP) geometry depicting the principal intensity-borrowing. Energies, the transition dipole moment ***μ***_S_3_–S_0__, and the spin–orbit coupling matrix element 〈S_3_|**H**_SO_|T_1_〉 were calculated at the TDA/(u)CAM-B3LYP-D3/6-311G(d) level. (b) Principal natural transition orbitals (NTOs) for S_3_–S_0_ (left) and T_1_–S_0_ (right) transitions.

**Fig. 6:**
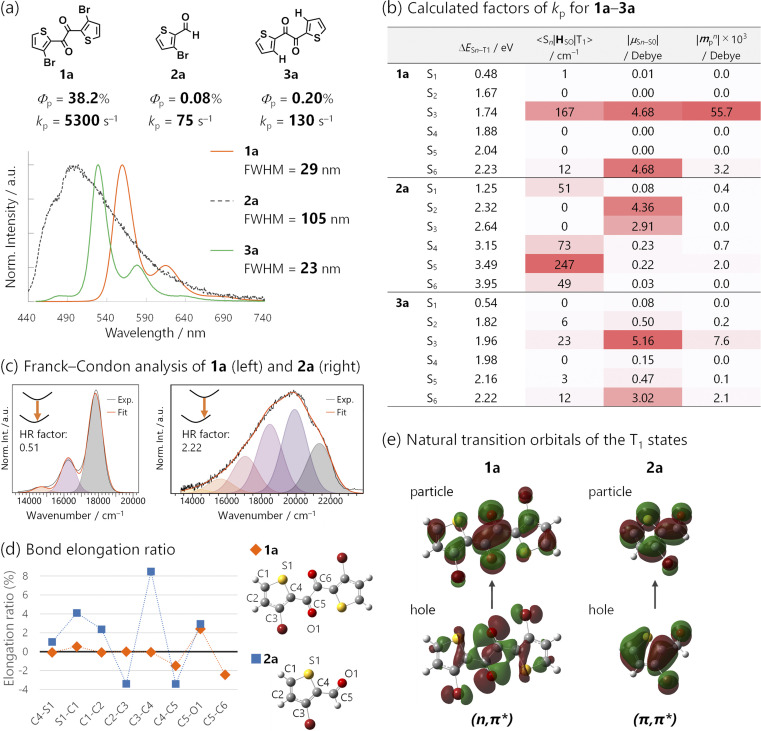
(a) Chemical structures of 1a, 2a, and 3a, and their *Φ*_p_, estimated *k*_p_, steady-state photoluminescence spectra, and the corresponding FWHM in cyclohexane (1.0 × 10^−5^ M, 2.6 × 10^−3^ M, and 1.0 × 10^−4^ M, respectively) excited at 368 nm under Ar. *k*_p_ was derived as *k*_p_ = *Φ*_p_/*τ*_p_, assuming *ϕ*_ISC_ = 1. (b) Calculated factors of *k*_p_ for 1a–3a. (c) Franck–Condon analysis of the emission spectra of 1a (left) and 2a (right). Huang–Rhys (HR) factors were 0.51 and 2.22, respectively. (d) Bond-elongation ratio between the optimized geometries in the S_0_ and T_1_ states of 1a and 2a. (e) Natural transition orbitals of the T_1_ states for 1a and 2a.

TDDFT calculations for the T_1_-optimized TP geometry of 1a yielded *k*_p_ = 5400 s^−1^ (for *n* = 1–6), which excellently reproduced the experimental value of 5300 s^−1^. Remarkably, the T_1_ state strongly coupled with the S_3_ state (Fig. 5a); a large SOC matrix element 〈S_3_|**H**_SO_|T_1_〉 = 167 cm^−1^ and a reasonable Δ*E*_S_3_–T_1__ = 1.74 eV = 14 000 cm^−1^ provided a large mixing coefficient 〈S_3_|**H**_SO_|T_1_〉/Δ*E*_S_3_–T_1__ of 1.19 × 10^−2^. This coefficient indicates that the T_1_ state can borrow 1.19% of the transition dipole moment between the S_3_ and S_0_ states (*i.e.*, ***μ***_S_3_–S_0__), which is quite large for phosphorescence. Furthermore, the |***μ***_S_3_–S_0__| was as large as 4.68 *D*, thus realizing a large |***m***_p_^3^| = 56 × 10^−3^*D*. The contribution from ***m***_p_^3^ corresponded to 93% of the total *k*_p_ value and is obviously the source of the large *k*_p_ (Fig. 6b, *vide infra*). Similar results were obtained for 1b, confirming that the origin of the substantial *k*_p_ lies in the thienyl diketone core (Table S5†).

The natural transition orbitals (NTOs) of the S_3_ and T_1_ states provided an intuitive understanding of the large ***μ***_S_3_–S_0__ and 〈S_3_|**H**_SO_|T_1_〉 (Fig. 5b). First, the S_3_ state (S_3_–S_0_ transition) had a pure (π,π*) configuration, where the NTOs delocalized over the entire molecule in a planar geometry, which was favourable for large ***μ***. On the other hand, the T_1_ state had a pure (n,π*) configuration, *i.e.*, it was not contaminated with (π,π*) character. The pure ^3^(n,π*) configuration enabled SOC with the pure ^1^(π,π*) state, consistent with El Sayed's rule.^4*a*^ The pure (n,π*)/(π,π*) characters are noteworthy, because excited states with mixed (n,π*) and (π,π*) configurations diminish SOC.^30^ Most importantly, the hole NTOs of the S_3_ and T_1_ states around the heavy atoms, especially Br, were mutually perpendicular. This is ideal because the spin–orbit Hamiltonian **H**_SO_ involves the orbital angular-momentum operator that rotates the orbitals by 90°.^13,28*a*^ Thus, for the hole-constituting atomic orbital of Br, the in-plane σ-symmetric n orbital of T_1_ states (Fig. 5b bottom-right) became π-symmetric, producing a significant spatial integral with the out-of-plane p orbital of the S_3_ state (Fig. 5b bottom-left). The large spatial integral near the heavy nuclei boosts the heavy-atom effect, resulting in a large 〈S_3_|**H**_SO_|T_1_〉 = 167 cm^−1^. Thus, 1 embodies an ideal electronic structure for intensity-borrowing that enhances *k*_p_: (1) a planar π-system that produces large ***μ***; (2) carbonyl groups in the π-plane that joined the pure ^1^(π,π*) and pure ^3^(n,π*) states; and (3) heavy atoms with electrons conjugated to both the π and σ-/n-systems to boost 〈S_*n*_|**H**_SO_|T_1_〉.

### Structure-RTP property relationship study

The significance of the diketone skeleton for the huge *k*_p_ was further evident when comparing 1 with the corresponding bromoaldehyde 2 and diketone without Br atoms 3 (Fig. 6a and b for 1a, 2a, and 3a; Fig. S5† for 1b, 2b, and 3b bearing triisopropylsilyl groups; also see Table S2†). All the compounds exhibited RTP in degassed solutions; however, *Φ*_p_ of 1 was much higher than those of 2 and 3 regardless of the silyl substituents (38.2%, 0.08%, and 0.20% for 1a, 2a, and 3a; 24.6%, 0.22%, and 1.2% for 1b, 2b, and 3b, respectively).

The superior *Φ*_p_ of 1 was attributed to its large *k*_p_, which was more than one order of magnitude larger than the estimated *k*_p_s of 2 and 3 (Fig. 6a and Table S2†). TDDFT calculations reasonably reproduced this trend; the calculated *k*_p_ for 1a, 2a, and 3a was 5400, 9, and 71 s^−1^, respectively (Fig. S27, S30 and S31†). The small *k*_p_ of aldehyde 2 was because of the mismatched 〈S_*n*_|**H**_SO_|T_1_〉 and ***μ***_S_*n*_–S_0__ (Fig. 6b; see Table S4† for 1b, 2b, and 3b). Thus, the T_1_ state of 2 had a (π,π*) configuration (Fig. 6e), which coupled strongly to (n,π*) S_*n*_ states (*e.g.*, *n* = 5; 〈S_5_|**H**_SO_|T_1_〉 was as large as 247 cm^−1^ for 2a). However, the (n,π*) configuration diminishes ***μ***_S_*n*_–S_0__ (*e.g.*, |***μ***_S_5_–S_0__| was only 0.22 *D* for 2a). Consequently, intensity-borrowing was insufficient, resulting in a small *k*_p_. Hence, a large 〈S_*n*_|**H**_SO_|T_1_〉 (or a large mixing coefficient, 〈S_*n*_|**H**_SO_|T_1_〉/Δ*E*_S_*n*_–T_1__) itself is insufficient and the combination with a large ***μ***_S_*n*_–S_0__ in the same *n*-th singlet state is essential for achieving a huge *k*_p_.^31^ To meet this requirement, the T_1_ state should have a (n,π*) configuration. Indeed, the *k*_p_ of Br-free diketone 3a was larger than that of Br-containing aldehyde 2a, because the T_1_ state of 3a had a (n,π*) configuration. Thus, the (n,π*) T_1_ state prefers coupling with (π,π*) S_*n*_ states, whose ***μ***_S_*n*_–S_0__ can be sizable (*e.g.*, *n* = 3, |***μ***_S_3_–S_0__| = 5.16 *D* for 3a; Fig. 6b). However, SOC between these states was not as effective as that for 1a because of the absence of the heavy atom, Br (*e.g.*, 〈S_3_|**H**_SO_|T_1_〉 = 23 cm^−1^ for 3a). Therefore, intensity borrowing was less effective, yielding a lower *k*_p_ than the brominated diketone 1a.

The side-on spatial arrangement of C–Br and C=O was crucial for the heavy-atom effect because of better mutual alignment of the in-plane Br and O p orbitals. This resulted in large hole coefficients in the T_1_ state (Fig. 5b).^32^ However, the arrangement is energetically unfavourable due to electronic repulsion, because Br and O in the TP geometry of 1 were within the sum of their van der Waals radii according to the X-ray structure (Fig. S32†).^17*a*,33,34^ Indeed, DFT calculations for the aldehyde 2a indicated that the side-on conformation was less stable (by 3 kcal mol^−1^) for both S_0_ and T_1_ states than the conformation with oxygen pointing away from the Br (Fig. S28†).^32^ Interestingly, the TP geometry of 1 involved two-fold intramolecular S⋯O noncovalent chalcogen bonding interactions,^35^ with interaction energies as large as 7.74 kcal mol^−1^, according to the natural bonding orbital analysis of 1a.^17*a*^ The chalcogen bonds stabilized the TP conformer and increased *k*_p_ by forcibly fixing Br in the vicinity of carbonyl oxygens.

### Origin of the narrowband emission

Finally, we investigated the origin of the narrowband emission of 1*via* a comparison of the 1a emission spectra with those of its aldehyde counterpart, 2a (Fig. 6a, c–e; see Fig. S18† for the comparison of 1b with 2b). Diketones 1a and 1b exhibited narrowband emissions (FWHM = 29 and 32 nm) while aldehydes 2a and 2b had broadband emissions (FWHM = 105 and 106 nm).

The bandwidths could be strongly correlated with molecular geometry changes in the ground and excited states, which are quantified using the Huang–Rhys (HR) factor obtained from a Franck–Condon analysis of the spectra (Fig. 6c, S18, and Table S3†).^15,36^ The HR factor of diketone 1a (1b) was 0.51 (0.50), while that of aldehyde 2a (2b) was 2.22 (2.39). The smaller HR factors in 1 corresponded to smaller geometry changes in the T_1_-to-S_0_ transition, which were corroborated by DFT calculations. The results indicated that although diketone 1a and aldehyde 2a retained their planarity in the transition, bond length changes were small for 1a and large for 2a.

We visualized these bond length changes using elongation ratios, defined as (*R*_T1_ − *R*_S_1__)/*R*_S_1__, where *R*_T_1__ and *R*_S_1__ represent bond lengths in the T_1_-and S_0_-optimized geometries; a positive value indicates that the bond is longer in the T_1_ state (Fig. 6d). The bond lengths within the thiophene ring of diketone 1a exhibited minimal changes. Conversely, the absolute values of the elongation ratios were large in the case of aldehyde 2a (*e.g.*, the bond length between 3C and 4C changed by 8.4%).

These considerable bond length changes in 2a were attributed to the (π,π*) character of the T_1_ state, as evident in the NTOs (Fig. 6e). The electronic transition from bonding to antibonding orbitals significantly affects the bond strength. In contrast, the NTOs of 1a were mainly localized on the 1,2-dicarbonyl moiety, and the electronic transition was nonbonding to antibonding, which led to minor changes in the bond lengths (Fig. 6d). Moreover, the transition in the diketones 1 had no net charge-transfer character because of the centrosymmetric geometry. This was in contrast to most aromatic (mono)carbonyl compounds, where ^3^(n,π*) states have a charge-transfer character from the carbonyl to the aromatic ring. These features of diketones minimized molecular geometry changes during phosphorescence emission. Therefore, the 1,2-diketone-based phosphor would be a promising platform for efficient narrowband RTP.

## Conclusions

Efficient narrowband RTP from metal-free organic 3-bromo-2-thienyl diketones was observed in solutions, amorphous polymer matrices, and crystalline solids. A substantial phosphorescence rate constant of ∼5000 s^−1^ was experimentally confirmed, which was the key to outstanding RTP quantum yields in solution (38% under Ar) and in polymer films (up to 54% in air). Ultrafast spectroscopy and single-crystal X-ray structure analysis revealed that the fast RTP originated from the diketone planar conformation. Both experimental and theoretical analyses indicated that the planar conformer embodies ideal electronic structures for fast RTP:

1. The planar geometry with a delocalized π-system

2. σ-Symmetric n-orbitals perpendicular to the π-system

3. Heavy atoms conjugated with both π- and n-electron systems

4. (n,π*) configuration of the T_1_ state

Fulfilling these points would be a promising design principle for molecules with large ***μ***_S_*n*_–S_0__ and 〈S_*n*_|**H**_SO_|T_1_〉 in the same S_*n*_ states, and results in a *k*_p_ leap. In addition, the centrosymmetric nonbonding-to-antibonding electronic transition of the 1,2-diketone skeleton was the key to the narrowband emission. The fast RTP (large *k*_p_) is potentially advantageous for various applications, such as phosphorescence OLEDs and lasers, triplet-to-singlet conversion *via* Förster resonance energy transfer, bioimaging, and theranostics. However, such applications have been monopolized by precious-metal phosphors. We believe that the mechanistic elucidation paves the way for developing fast organic RTP, which could expand applications of metal-free organic materials.

## Data availability

All experimental/computational procedures and data related to this article are provided in the ESI.†

## Author contributions

Y. T. project administration: lead; supervision: lead; conceptualisation: equal; funding acquisition: lead; investigation: lead; visualisation: lead; writing—original draft: lead; writing—review & editing: lead. K. M. project administration: lead; supervision: lead; conceptualisation: equal; funding acquisition: lead; investigation: supporting; visualisation: supporting; writing—original draft: supporting; writing—review & editing: lead. E. O., Y. O., M. T., and S. K. investigation: equal. M. K. investigation: equal; funding acquisition: supporting; writing—review & editing: supporting. T. E. and K. K. investigation: supporting. K. O. and T. O. resources: lead; supervision: supporting.

## Conflicts of interest

There are no conflicts to declare.

## Supplementary Material

SC-015-D4SC02841D-s001

SC-015-D4SC02841D-s002

SC-015-D4SC02841D-s003

SC-015-D4SC02841D-s004
