# Supplementary material for: Fast, efficient, narrowband room-temperature phosphorescence from metal-free 1,2-diketones: rational design and the mechanism
Source: Chem Sci. 2024 Jun 3;15(28):10784–93. doi: 10.1039/d4sc02841d (PMC11253173; doi:10.1039/d4sc02841d)
Supplement: SC-015-D4SC02841D-s002 [file SC-015-D4SC02841D-s002.pdf]

Electronic Supplementary Material (ESI) for Chemical Science.
This journal is © The Royal Society of Chemistry 2024

1a TP conformer @S_0

E(RB3LYP) -6478.74000672 a.u.

C	-1.4867050	4.0486100	0.0000000
C	-2.6588090	3.3442050	0.0000000
S	-2.4240920	1.6416790	0.0000000
C	-0.6743790	1.8358540	0.0000000
C	-0.3617230	3.1916670	0.0000000
C	0.2801340	0.7229600	0.0000000
O	1.4867050	0.8909240	0.0000000
C	-0.2801340	-0.7229600	0.0000000
O	-1.4867050	-0.8909240	0.0000000
C	0.6743790	-1.8358540	0.0000000
C	0.3617230	-3.1916670	0.0000000
C	1.4867050	-4.0486100	0.0000000
C	2.6588090	-3.3442050	0.0000000
S	2.4240920	-1.6416790	0.0000000
Br	1.3757560	3.9487050	0.0000000
Br	-1.3757560	-3.9487050	0.0000000
H	-1.4131550	5.1271940	0.0000000
H	-3.6619530	3.7482400	0.0000000
H	1.4131550	-5.1271940	0.0000000
H	3.6619530	-3.7482400	0.0000000

1a TP conformer @S_1

E(TD-HF/TD-DFT) -6478.65299649 a.u.

C	-1.4937870	4.0111770	0.0000000
C	-2.6662570	3.3070410	0.0000000
S	-2.4315710	1.5948950	0.0000000
C	-0.6841760	1.7994670	0.0000000
C	-0.3694140	3.1564300	0.0000000
C	0.2603240	0.7125130	0.0000000
O	1.4937870	0.8999840	0.0000000
C	-0.2603240	-0.7125130	0.0000000
O	-1.4937870	-0.8999840	0.0000000
C	0.6841760	-1.7994670	0.0000000
C	0.3694140	-3.1564300	0.0000000

C	1.4937870	-4.0111770	0.0000000
C	2.6662570	-3.3070410	0.0000000
S	2.4315710	-1.5948950	0.0000000
Br	1.3887780	3.8633960	0.0000000
Br	-1.3887780	-3.8633960	0.0000000
H	-1.4254600	5.0905270	0.0000000
H	-3.6721690	3.7023270	0.0000000
H	1.4254600	-5.0905270	0.0000000
H	3.6721690	-3.7023270	0.0000000

1a TP conformer @T_1

E(UB3LYP) -6478.66597736 a.u.

C	-1.4914120	4.0194440	0.0000000
C	-2.6642750	3.3185310	0.0000000
S	-2.4312750	1.6064920	0.0000000
C	-0.6837560	1.8076410	0.0000000
C	-0.3669200	3.1617700	0.0000000
C	0.2568680	0.7115080	0.0000000
O	1.4914120	0.8910470	0.0000000
C	-0.2568680	-0.7115080	0.0000000
O	-1.4914120	-0.8910470	0.0000000
C	0.6837560	-1.8076410	0.0000000
C	0.3669200	-3.1617700	0.0000000
C	1.4914120	-4.0194440	0.0000000
C	2.6642750	-3.3185310	0.0000000
S	2.4312750	-1.6064920	0.0000000
Br	1.3894040	3.8763550	0.0000000
Br	-1.3894040	-3.8763550	0.0000000
H	-1.4196000	5.0985110	0.0000000
H	-3.6698260	3.7146850	0.0000000
H	1.4196000	-5.0985110	0.0000000
H	3.6698260	-3.7146850	0.0000000

1b TP conformer @S_1

E(TD-HF/TD-DFT) -7768.01227585 a.u.

S	-2.8369220	0.0587510	0.6384550

C	-4.2670030	0.0005680	-0.3554360
C	-3.9262380	-0.0857220	-1.6892360
H	-4.6473350	-0.1370260	-2.4949580
C	-2.5354460	-0.1066360	-1.9147610
C	-1.7690010	-0.0352360	-0.7528830
C	-0.3308700	-0.0411850	-0.6818740
O	0.3897710	-0.1112200	-1.6971290
Br	-1.8357270	-0.2287970	-3.6753770
Si	-5.9999210	0.1341160	0.3882560
C	-5.9278510	-0.6426300	2.1276910
H	-5.0967400	-0.1112640	2.6147920
C	-5.5741780	-2.1401080	2.1409350
H	-5.3666180	-2.4777530	3.1621690
H	-4.6924270	-2.3666520	1.5365240
H	-6.3999730	-2.7491140	1.7642450
C	-7.1892610	-0.3722880	2.9678720
H	-8.0640340	-0.8856220	2.5579330
H	-7.4318390	0.6918910	3.0308770
H	-7.0563670	-0.7368880	3.9923450
C	-6.4250630	1.9885680	0.5439540
H	-7.4398580	2.0318790	0.9633730
C	-5.4834170	2.7239060	1.5135020
H	-4.4490720	2.7062970	1.1573110
H	-5.4927370	2.2897200	2.5170670
H	-5.7723700	3.7760440	1.6128580
C	-6.4489750	2.6918100	-0.8241870
H	-6.7025710	3.7518970	-0.7136850
H	-7.1830840	2.2535380	-1.5062630
H	-5.4707750	2.6426190	-1.3131560
C	-7.1811490	-0.6751450	-0.8725970
H	-6.9955610	-0.1122960	-1.7989000
C	-8.6651990	-0.4754570	-0.5146030
H	-9.3106740	-0.8465360	-1.3183790
H	-8.9200690	0.5753510	-0.3520960
H	-8.9369730	-1.0245410	0.3915640
C	-6.8859830	-2.1549620	-1.1731940

H	-7.1428930	-2.7949680	-0.3257000
H	-5.8337870	-2.3310480	-1.4097890
H	-7.4797180	-2.5008040	-2.0267520
S	2.8369220	-0.0587510	-0.6384550
C	4.2670030	-0.0005680	0.3554360
C	3.9262380	0.0857220	1.6892360
H	4.6473350	0.1370260	2.4949580
C	2.5354460	0.1066360	1.9147610
C	1.7690010	0.0352360	0.7528830
C	0.3308700	0.0411850	0.6818740
O	-0.3897710	0.1112200	1.6971290
Br	1.8357270	0.2287970	3.6753770
Si	5.9999210	-0.1341160	-0.3882560
C	7.1811490	0.6751450	0.8725970
H	6.9955610	0.1122960	1.7989000
C	6.8859830	2.1549620	1.1731940
H	7.4797180	2.5008040	2.0267520
H	5.8337870	2.3310480	1.4097890
H	7.1428930	2.7949680	0.3257000
C	8.6651990	0.4754570	0.5146030
H	8.9369730	1.0245410	-0.3915640
H	8.9200690	-0.5753510	0.3520960
H	9.3106740	0.8465360	1.3183790
C	6.4250630	-1.9885680	-0.5439540
H	7.4398580	-2.0318790	-0.9633730
C	6.4489750	-2.6918100	0.8241870
H	5.4707750	-2.6426190	1.3131560
H	7.1830840	-2.2535380	1.5062630
H	6.7025710	-3.7518970	0.7136850
C	5.4834170	-2.7239060	-1.5135020
H	5.7723700	-3.7760440	-1.6128580
H	5.4927370	-2.2897200	-2.5170670
H	4.4490720	-2.7062970	-1.1573110
C	5.9278510	0.6426300	-2.1276910
H	5.0967400	0.1112640	-2.6147920
C	7.1892610	0.3722880	-2.9678720

H	7.0563670	0.7368880	-3.9923450
H	7.4318390	-0.6918910	-3.0308770
H	8.0640340	0.8856220	-2.5579330
C	5.5741780	2.1401080	-2.1409350
H	6.3999730	2.7491140	-1.7642450
H	4.6924270	2.3666520	-1.5365240
H	5.3666180	2.4777530	-3.1621690

1b TP conformer @T_1

E(UB3LYP) -7768.02502842 a.u.

S	-2.8447000	0.0580360	0.6364500
C	-4.2750040	0.0011990	-0.3558660
C	-3.9342970	-0.0832110	-1.6883320
H	-4.6543360	-0.1336060	-2.4949920
C	-2.5415060	-0.1040010	-1.9144580
C	-1.7766210	-0.0339540	-0.7545120
C	-0.3342610	-0.0399600	-0.6775480
O	0.3895080	-0.1093870	-1.6907950
Br	-1.8506610	-0.2241280	-3.6798720
Si	-6.0082440	0.1342870	0.3884010
C	-5.9353590	-0.6422770	2.1278560
H	-5.1038320	-0.1112080	2.6145880
C	-5.5823890	-2.1399370	2.1411770
H	-5.3740900	-2.4774470	3.1623090
H	-4.7013440	-2.3671820	1.5359790
H	-6.4088310	-2.7486110	1.7653980
C	-7.1962590	-0.3713310	2.9686430
H	-8.0714750	-0.8841950	2.5590780
H	-7.4382700	0.6929700	3.0317590
H	-7.0630980	-0.7360030	3.9930550
C	-6.4330330	1.9886700	0.5437540
H	-7.4476850	2.0322380	0.9634700
C	-5.4910790	2.7242340	1.5128570
H	-4.4568720	2.7066540	1.1562310
H	-5.4999890	2.2902050	2.5165020
H	-5.7800300	3.7763720	1.6121530

C	-6.4573330	2.6916160	-0.8245410
H	-6.7105010	3.7518140	-0.7141510
H	-7.1919270	2.2534330	-1.5061390
H	-5.4794150	2.6419550	-1.3140150
C	-7.1893130	-0.6754090	-0.8719400
H	-7.0043550	-0.1123220	-1.7982300
C	-8.6734300	-0.4768190	-0.5135820
H	-9.3188790	-0.8478960	-1.3173630
H	-8.9288970	0.5737700	-0.3505110
H	-8.9446800	-1.0264900	0.3923950
C	-6.8932340	-2.1549690	-1.1731020
H	-7.1494950	-2.7954710	-0.3257860
H	-5.8410010	-2.3302080	-1.4101420
H	-7.4869470	-2.5008880	-2.0266300
S	2.8447000	-0.0580360	-0.6364500
C	4.2750040	-0.0011990	0.3558660
C	3.9342970	0.0832110	1.6883320
H	4.6543360	0.1336060	2.4949920
C	2.5415060	0.1040010	1.9144580
C	1.7766210	0.0339540	0.7545120
C	0.3342610	0.0399600	0.6775480
O	-0.3895080	0.1093870	1.6907950
Br	1.8506610	0.2241280	3.6798720
Si	6.0082440	-0.1342870	-0.3884010
C	7.1893130	0.6754090	0.8719400
H	7.0043550	0.1123220	1.7982300
C	6.8932340	2.1549690	1.1731020
H	7.4869470	2.5008880	2.0266300
H	5.8410010	2.3302080	1.4101420
H	7.1494950	2.7954710	0.3257860
C	8.6734300	0.4768190	0.5135820
H	8.9446800	1.0264900	-0.3923950
H	8.9288970	-0.5737700	0.3505110
H	9.3188790	0.8478960	1.3173630
C	6.4330330	-1.9886700	-0.5437540
H	7.4476850	-2.0322380	-0.9634700

C	6.4573330	-2.6916160	0.8245410
H	5.4794150	-2.6419550	1.3140150
H	7.1919270	-2.2534330	1.5061390
H	6.7105010	-3.7518140	0.7141510
C	5.4910790	-2.7242340	-1.5128570
H	5.7800300	-3.7763720	-1.6121530
H	5.4999890	-2.2902050	-2.5165020
H	4.4568720	-2.7066540	-1.1562310
C	5.9353590	0.6422770	-2.1278560
H	5.1038320	0.1112080	-2.6145880
C	7.1962590	0.3713310	-2.9686430
H	7.0630980	0.7360030	-3.9930550
H	7.4382700	-0.6929700	-3.0317590
H	8.0714750	0.8841950	-2.5590780
C	5.5823890	2.1399370	-2.1411770
H	6.4088310	2.7486110	-1.7653980
H	4.7013440	2.3671820	-1.5359790
H	5.3740900	2.4774470	-3.1623090

2a s-*cis* @S_0

E(RB3LYP) -3239.96819212 a.u.

C	2.2281710	1.0950300	0.0000000
C	0.9466190	1.5745600	0.0000000
C	0.0000000	0.5191580	0.0000000
C	0.5601560	-0.7410160	0.0000000
S	2.3031300	-0.6265520	0.0000000
H	0.6819290	2.6229180	0.0000000
C	-0.0861650	-2.0518720	0.0000000
O	0.5138420	-3.1053340	0.0000000
H	-1.1922410	-2.0193090	0.0000000
Br	-1.8709840	0.8632930	0.0000000
H	3.1412550	1.6734860	0.0000000

2a s-*trans* @S_0

E(RB3LYP) -3239.96310971 a.u.

C	2.0011990	1.6214110	0.0000000

C	0.6383140	1.7314140	0.0000000
C	0.0000000	0.4642630	0.0000000
C	0.8754590	-0.6028170	0.0000000
S	2.5283740	-0.0172550	0.0000000
H	0.1013240	2.6701400	0.0000000
C	0.6318670	-2.0494880	0.0000000
O	-0.4573580	-2.5733170	0.0000000
H	1.5580280	-2.6649460	0.0000000
Br	-1.8874860	0.3270420	0.0000000
H	2.7265160	2.4222470	0.0000000

2a s-*cis* @T_1

E(UB3LYP) -3239.87130595 a.u.

C	2.2707620	1.0208590	0.0000000
C	0.9779540	1.5600900	0.0000000
C	0.0000000	0.6013000	0.0000000
C	0.5304260	-0.7974550	0.0000000
S	2.2950590	-0.7728750	0.0000000
H	0.7802760	2.6242720	0.0000000
C	-0.1610830	-2.0280610	0.0000000
O	0.4357890	-3.1241470	0.0000000
H	-1.2614670	-1.9791130	0.0000000
Br	-1.8467690	0.9430530	0.0000000
H	3.2024850	1.5667760	0.0000000

2a s-*trans* @T_1

E(UB3LYP) -3239.86584717 a.u.

C	2.0107570	-1.5956050	0.1698430
C	0.6329930	-1.6993230	0.2724440
C	-0.0289670	-0.4958110	0.1169030
C	0.9047540	0.6726840	-0.0252410
S	2.5556400	0.0790390	-0.2126800
H	0.1237630	-2.6405360	0.4354560
C	0.6224910	2.0591360	0.0782220
O	-0.5143280	2.5253220	0.2474930
H	1.4978740	2.7296430	-0.0178550

Br	-1.8856090	-0.3665590	-0.0850940
H	2.7469080	-2.3732270	0.3106000

2b s-*cis* @T_1

E(UB3LYP) -3884.55328910 a.u.

C	0.0816690	0.1408480	-0.0040660
C	-0.9340640	-0.8449380	0.0485640
C	-2.2072610	-0.3567900	-0.0100170
C	-2.2850060	1.1266490	-0.1139120
S	-0.6592400	1.7948250	-0.1387170
H	-0.7133860	-1.9022530	0.1250510
C	-3.4162560	1.9707830	-0.1852180
O	-3.3193970	3.2109900	-0.2714530
H	-4.4022710	1.4789910	-0.1617960
Br	-3.7641150	-1.4173640	0.0278900
Si	1.9450380	-0.0924180	0.0972610
C	2.7170330	1.1963140	-1.0764500
H	2.3581650	2.1560370	-0.6767120
C	2.4985960	0.2728370	1.8906000
H	3.5786510	0.0700660	1.9101880
C	2.2760730	-1.9316350	-0.2896730
H	1.6118290	-2.4695200	0.4025230
C	4.2545380	1.2232100	-1.0133260
H	4.6510110	2.0597870	-1.5988710
H	4.6317410	1.3335040	0.0073060
H	4.6892240	0.3093650	-1.4290600
C	2.2262260	1.1053770	-2.5320040
H	1.1355810	1.0946520	-2.6025900
H	2.5823810	1.9641280	-3.1112920
H	2.5994250	0.2065930	-3.0288210
C	1.8208130	-0.6720620	2.8980480
H	2.0178800	-1.7260670	2.6824450
H	2.1773790	-0.4777410	3.9154570
H	0.7350340	-0.5328430	2.9035640
C	2.2893250	1.7418570	2.2967490
H	2.8177030	2.4369310	1.6387920

H	1.2306190	2.0167930	2.2857850
H	2.6570260	1.9171670	3.3138280
C	3.7147520	-2.3701360	0.0406770
H	3.9912560	-2.1542010	1.0758240
H	3.8329920	-3.4489340	-0.1090010
H	4.4459650	-1.8761760	-0.6054190
C	1.8937670	-2.3577470	-1.7181320
H	1.9319120	-3.4478910	-1.8200970
H	0.8879870	-2.0347790	-1.9999680
H	2.5871750	-1.9445480	-2.4548830

3a @T_1

E(UB3LYP) -1331.58223165 a.u.

C	0.0274000	4.4106700	-0.0559520
C	1.2557540	3.8506880	-0.2928250
C	1.2313820	2.4361060	-0.2852540
C	-0.0274000	1.9263510	-0.0135570
S	-1.1997630	3.2185330	0.1874940
H	-0.2265700	5.4598930	-0.0093130
H	2.1515430	4.4324500	-0.4702230
H	2.0918320	1.8118850	-0.4709190
C	-0.5014010	0.5600230	0.0814130
O	-1.7199860	0.3002800	0.1684500
C	0.5014010	-0.5600230	0.0814130
O	1.7199860	-0.3002800	0.1684500
C	0.0274000	-1.9263510	-0.0135570
C	-1.2313820	-2.4361060	-0.2852540
S	1.1997630	-3.2185330	0.1874940
C	-1.2557540	-3.8506880	-0.2928250
H	-2.0918320	-1.8118850	-0.4709190
C	-0.0274000	-4.4106700	-0.0559520
H	-2.1515430	-4.4324500	-0.4702230
H	0.2265700	-5.4598930	-0.0093130

3b @T_1

E(UB3LYP) -2620.93887232 a.u.

O	-0.4059800	-1.6687980	-0.3461300
C	-0.5888980	-0.4547680	-0.1137910
C	-1.9224480	0.0856120	0.0556830
C	-2.3530610	1.3316130	0.4845980
H	-1.6772020	2.1250910	0.7650490
C	-3.7603270	1.4375070	0.5058740
H	-4.2695080	2.3497370	0.7941010
C	-4.4372440	0.2912750	0.1284820
S	-3.2926240	-0.9601040	-0.2675450
Si	-6.3027530	0.0030450	0.0952650
C	-7.1072340	1.7171200	-0.1367880
H	-6.7145500	2.3022260	0.7074900
C	-6.8172330	-0.7021800	1.7941520
H	-7.9057180	-0.8453520	1.7462210
C	-6.6516120	-1.3007050	-1.2523780
H	-5.9868650	-2.1351050	-0.9834980
C	-8.6394650	1.6880740	0.0099510
H	-9.1106260	1.1368740	-0.8090250
H	-8.9631860	1.2262050	0.9467660
H	-9.0503110	2.7035990	-0.0111660
C	-6.6963720	2.4502010	-1.4258380
H	-7.0456930	3.4887120	-1.4071200
H	-5.6130480	2.4681720	-1.5676590
H	-7.1352680	1.9822610	-2.3103700
C	-6.5193660	0.2801670	2.9399360
H	-5.4486720	0.4979120	3.0071620
H	-7.0443260	1.2321830	2.8203900
H	-6.8262680	-0.1397410	3.9043180
C	-6.1743590	-2.0713680	2.0754020
H	-6.5111230	-2.4688420	3.0393510
H	-6.4238360	-2.8154070	1.3137910
H	-5.0834860	-1.9989140	2.1199950
C	-8.0919220	-1.8429280	-1.2192530
H	-8.2120850	-2.6660030	-1.9323460
H	-8.3748780	-2.2229770	-0.2339750
H	-8.8184940	-1.0732980	-1.4958330

C	-6.2678210	-0.8599150	-2.6758470
H	-6.9534970	-0.0986360	-3.0566910
H	-5.2560940	-0.4501620	-2.7278140
H	-6.3144220	-1.7078440	-3.3680580
O	0.4050460	1.7074300	0.0276170
C	0.5886590	0.4721490	-0.0146860
C	1.9230390	-0.0912160	0.0212590
C	2.3555940	-1.4000420	0.1694660
H	1.6809780	-2.2354870	0.2788570
C	3.7629280	-1.5064060	0.1630910
H	4.2736600	-2.4580680	0.2522970
C	4.4381750	-0.3048010	0.0415520
S	3.2917500	1.0011210	-0.0723000
Si	6.3014130	-0.0265570	-0.0820560
C	6.4536540	-1.3670040	-2.6101920
H	6.7381320	-1.3484360	-3.6681250
H	6.9841300	-2.2042930	-2.1477640
H	5.3824950	-1.5881860	-2.5664420
C	6.7736790	-0.0234210	-1.9326020
H	7.8624860	0.1212520	-1.9671090
C	6.6669280	1.6925070	0.6585760
H	5.9949560	2.3613480	0.1003250
C	8.1044710	2.1771980	0.3981020
H	8.8376460	1.5712240	0.9384270
H	8.3716970	2.1486890	-0.6616730
H	8.2322250	3.2108100	0.7382820
C	6.3055130	1.8323930	2.1477050
H	6.3599370	2.8808120	2.4611180
H	5.2956490	1.4770520	2.3672080
H	6.9984150	1.2731800	2.7816430
C	6.1204240	1.1385800	-2.7008090
H	5.0291420	1.0605080	-2.6917400
H	6.3841080	2.1153950	-2.2858090
H	6.4352740	1.1367140	-3.7502870
C	7.1264090	-1.5229330	0.7667020
H	6.7148310	-2.3861000	0.2237310

C	6.7586610	-1.7027200	2.2500060
H	7.2187980	-0.9318740	2.8730330
H	5.6800010	-1.6610530	2.4199130
H	7.1160230	-2.6696160	2.6219230
C	8.6535430	-1.5578350	0.5750910
H	9.0726900	-2.4895210	0.9711190
H	8.9462540	-1.4911570	-0.4762970
H	9.1439710	-0.7369490	1.1063320
