# Supplementary material for: Fast, efficient, narrowband room-temperature phosphorescence from metal-free 1,2-diketones: rational design and the mechanism
Source: Chem Sci. 2024 Jun 3;15(28):10784–93. doi: 10.1039/d4sc02841d (PMC11253173; doi:10.1039/d4sc02841d)
Supplement: SC-015-D4SC02841D-s003 [file SC-015-D4SC02841D-s003.pdf]

Electronic Supplementary Material (ESI) for Chemical Science.
This journal is © The Royal Society of Chemistry 2024

Supporting Information

for

Fast, Efficient, Narrowband Room-Temperature Phosphorescence from Metal-Free 1,2-Diketones: Rational Design and Mechanism

Yosuke Tani,* Kiyoshi Miyata,* Erika Ou, Yuya Oshima, Mao Komura, Morihisa Terasaki, Shuji Kimura, Takumi Ehara, Koki Kubo, Ken Onda and Takuji Ogawa

Department of Chemistry, Graduate School of Science, Osaka University Machikaneyama 1-1, Toyonaka, Osaka 560-0043, Japan
Innovative Catalysis Science Division, Institute for Open and Transdisciplinary Research Initiatives (ICS-OTRI), Osaka University, Suita, Osaka 560-8531, Japan
Department of Chemistry, Faculty of Science, Kyushu University, 744 Motooka, Nishi, Fukuoka 819-0395, Japan
E-mail: y-tani@chem.sci.osaka-u.ac.jp; kmiyata@chem.kyushu-univ.jp

Table of Contents

1. Literature Survey on Organic RTP Properties in Solution 2
2. Instrumentation and Chemicals 4
3. Synthesis Procedures 5
4. Photophysical Properties.. 7
5. Theoretical Calculations 26
6. Single-Crystal X-ray Structure Analysis... 31
7. NMR Charts.. 34
8. References... 39

1. Literature Survey on Organic RTP Properties in Solution

Table S1 lists the RTP properties of representative metal-free organic phosphors in solution (Figure S1), where RTP quantum yields Φ_p and lifetimes τ_p are available and Φ_p >0.3%. Note that Φ_p may contain a fluorescence component. Full-width-at-half-maxima (FWHM) was manually red from the reported spectra. The phosphorescence rate constants (k_p) and the nonradiative decay rate constants from the triplet state (k_{nr}) were derived according to $k_p = \Phi_p / \tau_p$ and $k_{nr} = (1-\Phi_p) / \tau_p$. Here we assumed the quantum yield of intersystem crossing $\phi_{ISC} = 1$ for all cases, which may cause an error. Also, the measurement of Φ_p and τ_p may be conducted with different degrees of degassing and/or excitation wavelengths, which can be another source of error. Accordingly, the values listed in Table S1 and Figure 1 should be considered as a guide for comparison.

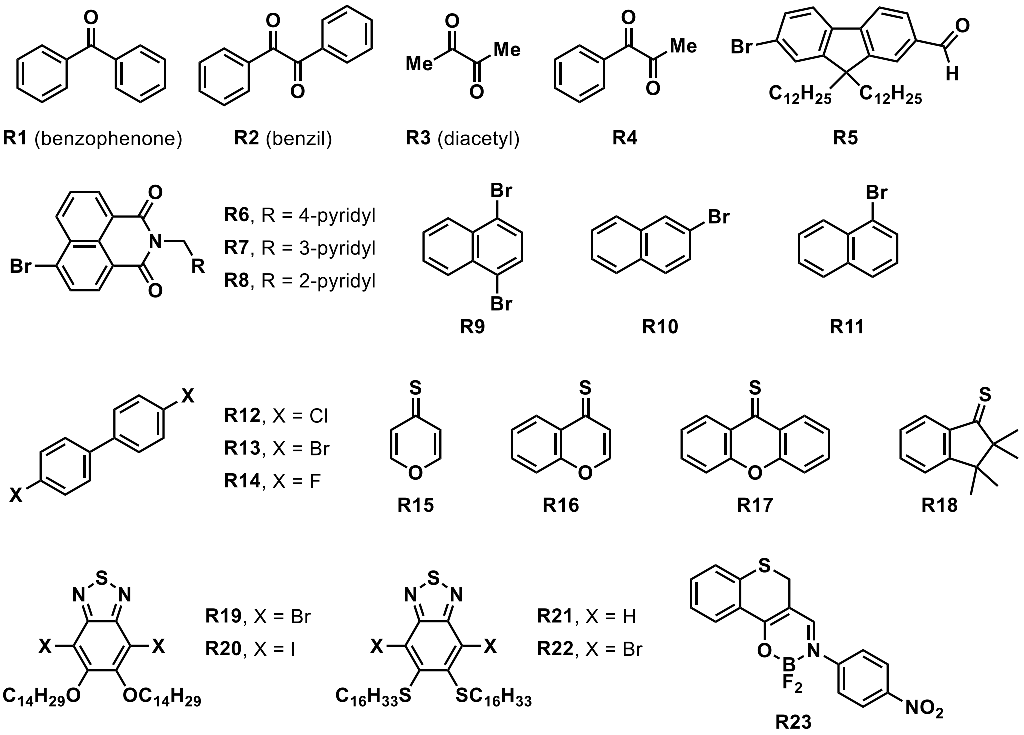

Figure S1. Chemical structures of representative metal-free organic phosphors exhibiting RTP in solution.

Table S1. Summary of Reported Organic RTP Properties in Solution

	Φ_p (%)	τ_p / μs	FWHM / nm	k_p /s^{-1}	k_{nr} /s^{-1}	ref
**R1**	1	50	87	200	20000	[1]
**R2**	0.31	80	41	39	12000	[2]
**R3**	2.8	270	22	100	3600	[3]
**R4**	1.2	160	28	75	6200	[3]
**R5**	5.9	355	82	170	2700	[4]
**R6**	0.59	332	116	18	3000	[5]
**R7**	0.56	317	114	18	3100	[5]
**R8**	0.46	287	113	16	3500	[5]
**R9**	18	1700	81	110	480	[6]
**R10**	14	2800	105	50	310	[6]
**R11**	10	1900	—	53	470	[6]
**R12**	12	1700	116	71	520	[6]
**R13**	8	860	—	93	1100	[6]
**R14**	5	850	—	59	1100	[6]
**R15**	3.4	6.5	71	5200	150000	[7]
**R16**	3.2	7.3	—	4400	130000	[8]
**R17**	3.5	7.1	—	4900	140000	[8]
**R18**	6.9	30	—	2300	31000	[8]
**R19**	(0.55)^a	2.8	n.d.^{b}			[9]
**R20**	(0.59)^a	3.6	n.d.^{b}			[9]
**R21**	(0.7)^a	5.4	n.d.^{b}			[9]
**R22**	(0.5)^a	3.4	n.d.^{b}			[9]
**R23**	10	(8.3–25)^c	89			[10]

^{a}Reported to be determined by a relative method although the spectra were end at ~720 nm (along the middle of the red RTP). ^{b}Not determined due to the reason noted in *a*. ^{c}Two different measurements provided significantly different triplet lifetime.

2. Instrumentation and Chemicals

Unless otherwise noted, all the reactions were performed under a nitrogen or argon atmosphere using anhydrous solvents and heat-gun-dried glassware on a dual-manifold Schlenk line. ^{1}H and ^{13}C{^{1}H} NMR spectra were recorded on a JEOL ECS400 or ECA500 spectrometer. Chemical shift values (δ) are reported in ppm and are calibrated to tetramethylsilane (0.00 ppm) or residual solvent (7.26 ppm in $CDCl_3$) for ^{1}H, and to $CDCl_3$ (77.0 ppm) for ^{13}C NMR. Melting points were measured in a glass capillary with a Barnstead/Thermolyne Mel-Temp melting point apparatus or using a Hitachi NEXTA DSC200. High-resolution mass spectra (ESI-HRMS) were obtained with a Thermo Fisher Scientific LTQ Orbitrap XL mass spectrometer. Elemental analysis (EA) was conducted on a Yanaco MT-5 or MT-6 recorder. UV-vis absorption and steady-state photoluminescence (PL) spectra, PL quantum yields (PLQY, Φ_{PL}), and PL lifetimes were measured at room temperature (RT). UV-vis absorption spectra in solutions were obtained using a Shimadzu UV-3150, a JASCO V-750, or a Hitachi U-4100 spectrometer. Photographs were taken using SONY NEX-5N under incandescent light or under irradiation with UV light (365 nm). Analytical thin-layer chromatography (TLC) was performed on aluminum plates bearing a layer of Merck silica gel 60 F_{254}. Column chromatography was carried out on silica-gel 60 or 60N (Kanto Chemical Co., Inc., spherical, 63–210 μm).

Anhydrous THF was purchased from Wako Chemical Co., Inc., and further purified by passage through activated alumina under positive nitrogen pressure as described by Grubbs et al.[11] 3-Bromothiophene-2-carbaldehyde (**2a**) was purchased from Tokyo Chemical Industry Co., Ltd., and further purified by gel permeation chromatography using a JAI LC-9130 NEXT equipped with JAIGEL-1HR and 2HR (eluent: $CHCl_3$, flow rate: 10 mL/min). 1,2-di(thiophen-2-yl)ethane-1,2-dione (**3a**) was purchased from Tokyo Chemical Industry Co., Ltd., and further purified by recrystallization from MeOH. Unless otherwise noted, chemicals obtained from commercial suppliers were used without further purification. 2-(Pentafluorophenyl)-6,7-dihydro-5*H*-pyrrolo[2,1-*c*][1,2,4]triazol-2-ium tetrafluoroborate (**S1**), 1,2-bis[3-bromothiophen-2-yl]ethane-1,2-dione (**1a**), 1,2-bis[3-bromo-5-(triisopropylsilyl)thiophen-2-yl]ethane-1,2-dione (**1b**), 3-bromo-5-(triisopropylsilyl)thiophene-2-carbaldehyde (**2b**), and 1,2-bis[5-(triisopropylsilyl)thiophen-2-yl]ethane-1,2-dione (**3b**) were synthesized according to our previous report.[12] **2b** was further purified by vacuum sublimation before photophysical measurements.

3. Synthesis Procedures

Scheme S1. Synthesis of 1c

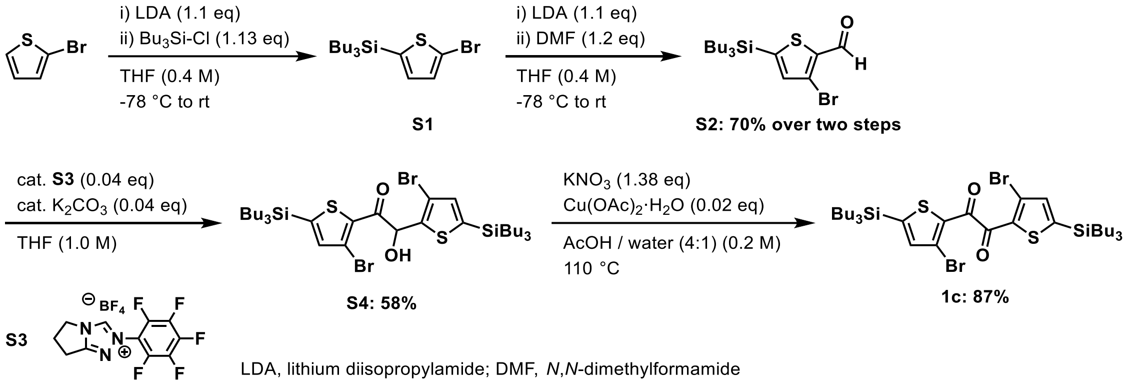

3-bromo-5-(tributylsilyl)thiophene-2-carbaldehyde (S2)

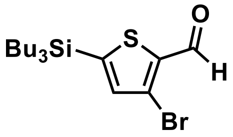

S2

To a 50 mL 2-neck flask were added diisopropylamine (2.5 mL, 17.7 mmol) and anhydrous THF (10 mL). The flask was cooled to –30 °C and *n*-butyllithium (BuLi, 2.80 M in hexane, 6.0 mL, 16.8 mmol) was added dropwise. The mixture was stirred for 20 min at 0 °C to prepare LDA solution. To another 200 mL 2-neck flask were added 2-bromothiophene (1.55 mL, 16.0 mmol) and THF (30 mL). The flask was cooled to –78 °C and the freshly prepared LDA solution (cooled to –78 °C) was added dropwise via cannula. The mixture was stirred for 30 min at the same temperature before chlorotributylsilane (4.8 mL, 18.0 mmol) was added dropwise. After stirring the mixture for 1 h at –78 °C, the cooling bath was removed and the mixture was stirred overnight at RT. The reaction was quenched by adding aq. NH_4Cl and Et_2O. The organic layer was separated and the aqueous layer was extracted with Et_2O. The combined organic extracts were washed with brine, dried over $MgSO_4$, filtered, and then evaporated. The residue was purified by silica-gel column chromatography (eluent: hexane) to give 2-bromo-5-(tributylsilyl)thiophene **S1** as an orange oil. To another 200 mL 2-neck flask were added **S1** (5.0 g, 13.8 mmol) and THF (27 mL). LDA was again prepared by the same method from diisopropylamine (2.2 mL, 15.6 mmol), BuLi (2.69 M in hexane, 5.4 mL, 14.5 mmol), and THF (8 mL). To the **S1** solution was transferred LDA dropwise via cannula at –78 °C. The resulting mixture was stirred for 2 h, and then DMF (1.3 mL, 16.8 mmol) was added dropwise. After 15min, the cooling bath was removed and the mixture was stirred overnight at RT. The reaction was quenched by adding aq. NH_4Cl and Et_2O. The organic layer was separated and the aqueous layer was extracted with Et_2O. The combined organic extracts were washed with brine, dried

over $MgSO_4$, filtered, and then evaporated. The residue was purified by silica-gel column chromatography (eluent: hexane to hexane/CH_2Cl_2 2:1) to give **S2** (4.35 g, 70%) as an orange oil. **^{1}H NMR** (400 MHz, $CDCl_3$) δ: 9.95 (s, 1H), 7.19 (s, 1H), 1.40–1.26 (m, 12H), 0.89, (t, J = 7.0 Hz, 9H), 0.82 (m, 6H). **^{13}C NMR** (125 MHz, $CDCl_3$) δ: 182.6 (CH), 150.4 (Cq), 140.6 (Cq), 138.0 (CH), 120.7 (Cq), 26.5 (CH_2), 25.7 (CH_2), 13.6 (CH_3), 12.6 (CH_2). **ESI-HRMS** (*m*/*z*): $[M+H]^+$ calcd for $C_{17}H_{30}BrOSSi$, 389.0965; found, 389.0965.

1,2-bis[3-bromo-5-(tributylsilyl)thiophen-2-yl]ethane-1,2-dione (1c)

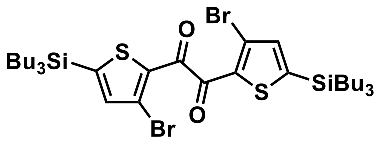

1c

To a Schlenk tube were added K_2CO_3 (61.7 mg, 0.446 mmol) and triazolium salt **S3** (162 mg, 0.446 mmol), and the tube was shortly evacuated and backfilled with N_2 three times. THF (6 mL) was added via a syringe and the suspension was stirred for 15 minutes. To the pale red suspension were added **S2** (4.35 g, 11.16 mmol) under a flow of N_2. The mixture was stirred for 48 h at RT, and then quenched by adding aq. NH_4Cl and CH_2Cl_2. The organic layer was separated and the aqueous layer was extracted with CH_2Cl_2. The combined organic extracts were dried over $MgSO_4$, filtered, and then evaporated. The residue was purified by silica-gel column chromatography (eluent: hexane/CH_2Cl_2 2:1) to give **S4** (2.52 g, 58%) as an orange liquid. **^{1}H NMR** (400 MHz, $CDCl_3$) δ: 7.13 (s, 1H), 7.03 (s, 1H), 6.10 (d, J = 6.2 Hz, 1H), 4.42 (d, J = 6.2 Hz, 1H), 1.34–1.27 (m, 24H), 0.90–0.83 (m, 18H), 0.78–0.70 (m, 12H).

To a Schlenk tube were added **S4** (2.52 g, 3.24 mmol), KNO_3 (452 mg, 4.47 mmol), $Cu(OAc)_2{\cdot}H_2O$ (12.9 mg, 65 μmol), and AcOH/H_2O (4:1, 16 mL). The mixture was stirred overnight at 110 °C. After cooling to RT, the mixture was quenched by adding H_2O and CH_2Cl_2. The organic layer was separated and the aqueous layer was extracted with CH_2Cl_2 three times. The residue was purified by silica-gel column chromatography three times (eluent: hexane/AcOEt 30:1 for the first run, hexane/CH_2Cl_2 2:1 for the second run, and hexane/CH_2Cl_2 4:1 for the third run) to give **1c** (2.18 g, 87%) as an orange liquid. **m.p.** 79.2 °C. **^{1}H NMR** (400 MHz, $CDCl_3$) δ: 7.22 (s, 1H), 1.40–1.27 (m, 12H), 0.89 (t, J = 6.9 Hz, 9H), 0.85–0.81 (m, 6H). **^{13}C NMR** (100 MHz, $CDCl_3$) δ: 182.36, 152.45, 139.33, 135.95, 119.93, 26.51, 25.73, 13.65, 12.55. **EA** Calcd for $C_{34}H_{56}Br_2O_2S_2Si_2$: C, 52.56; H, 7.27. Found: C, 52.54; H, 7.21.

4. Photophysical Properties

Typical procedure for evaluating PLQY and lifetime in solution

The protocol was summarized in Scheme S2. To a quartz cell (path length: 10 mm) with extended tube at the top (ϕ = 8 mm, l = 55 mm) was added a solution of **1a** in spectral-grade cyclohexane (1.0×10^{-4} M). The UV-vis absorption spectrum was acquired using a Shimadzu UV-3150 spectrometer. Then, the solution was changed to 1.0×10^{-5} M, and the UV-vis absorption and steady-state PL spectra in air (λ_{ex} = 368 nm) were acquired using a Shimadzu UV-3150 and a JASCO FP-8200 spectrometer, respectively. After that, PL decay curve at 560 nm in air was acquired using a HORIBA DeltaFlex multichannel scaling system using DeltaDiode for excitation (368 nm). Note that we used the same excitation wavelength for measuring PL spectra and decay curves, so that we can eliminate any excitation-wavelength dependence (if any). Next, the solution was degassed by bubbling Ar for approximately 5 minutes using a Pasteur pipette and the cell was capped with a rubber septum. The steady-state PL spectrum and PL decay curve were sequentially acquired. Subsequently, the steady-state PL spectrum was measured again to confirm the negligible change in the PL intensity, thus ensuring that the degree of degassing was kept for the PL and lifetime measurements. In addition, the UV-vis absorption spectrum was measured, confirming the negligible change in absorbance (concentration). Based on thus obtained PL spectra, PL quantum yields ($\Phi_{PL} \sim \Phi_p$, see the main text for detail) in air and under Ar were determined to be 2.6 and 38.2%, respectively, by the relative method using quinine sulfate as the standard (60% in 0.5 M H_2SO_4 aq.).[13] The lifetimes τ_p in air and under Ar were obtained as 4.83±0.015 and 72.7±0.12 μs, respectively, from a single-exponential fit to the decay curve using a HORIBA EzTime software. Finally, based on the obtained Φ_p and τ_p, the rate constants k_p and k_{nr} were derived according to the formulas $k_p = \Phi_p / \tau_p$ and $k_{nr} = (1-\Phi_p) / \tau_p$. Here we applied $\phi_{ISC} = 1$ as we confirmed in the main text. We judged that the data were valid when the difference in k_p in air and under Ar was less than 5%, further assuring the consistent degassing for the measurement of Φ_p and τ_p.

We would like to emphasize that, in literatures, it is often not explicitly addressed or stated whether the degree of the degassing is consistent when measuring Φ_p and τ_p. However, if there is a difference in the degree of degassing, it is imperative to acknowledge that determining rate constants from these values would yield fundamentally flawed outcomes. Indeed, in our previous report,[12] Φ_p and τ_p of **1b** in cyclohexane were reported to be 13% and 37 μs at RT (Figure S2). However, the degree of degassing is not assured. Consequently, it provided much different k_p if we calculate Φ_p / τ_p: 3,500 s^{-1}. Note that the smaller Φ_p and shorter τ_p were due to the less efficient degassing; we used nitrogen instead of argon and quartz cell without extended tube in the previous report.

Scheme S2. Protocol of Photophysical Property Measurements for k_p Determination

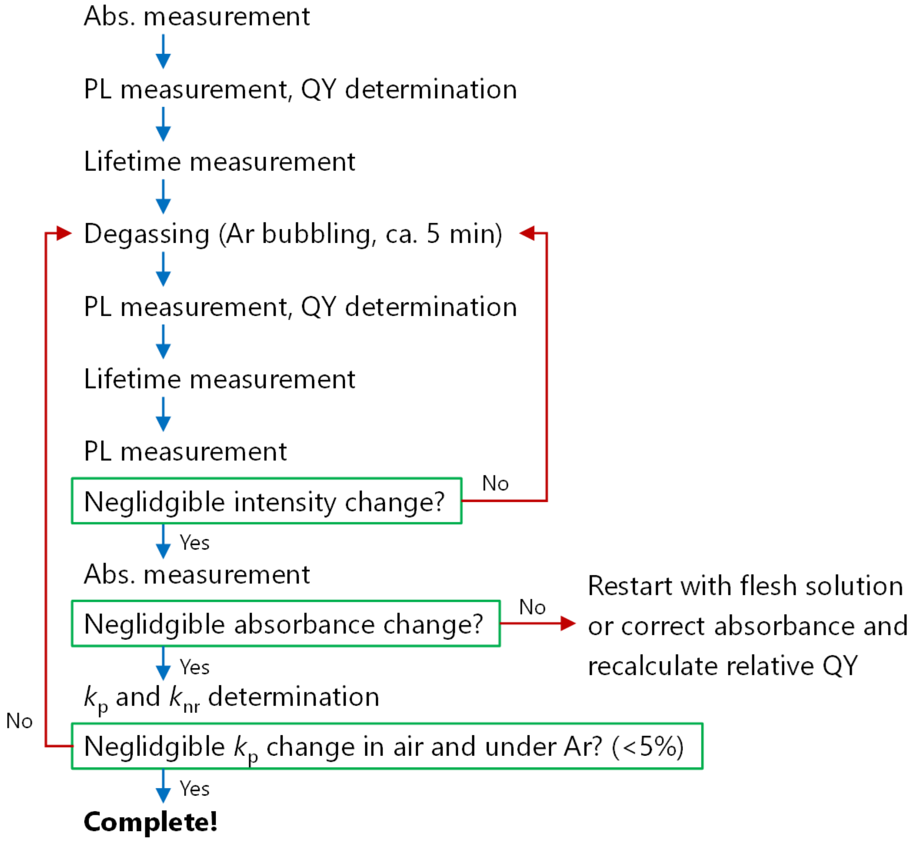

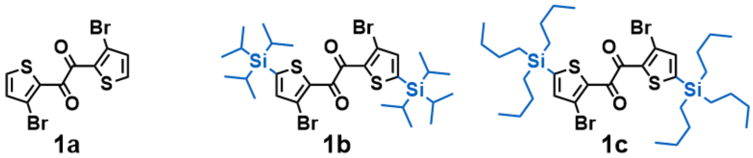

	1a		1b		1c	
	Φ_p (%)	τ_p / μs	Φ_p (%)	τ_p / μs	Φ_p (%)	τ_p / μs
This Work	38.2	72.7	24.6	49.3	17.0	37.7
Previous Work (*J. Mater. Chem. C* **2019**)	22	n.d.*	13#	37#	(not synthesized)	

*n.d. = not determined. #These values were obtained with independently degassed sample solutions; therefore, the oxygen concentrations of the samples were different each other.

Figure S2. Comparison of RTP properties of **1a–1c** in solution reported in previous work and present work.

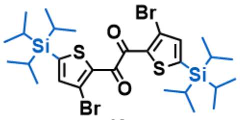

1b

Mechanochromic RTP

J. Mater. Chem. C **2019**, *7*, 11926–11931.

Properteis in solution

✓ PL spectrum, PLQY, lifetime

☐ Inconsistent degassing

☐ ISC not evaluated

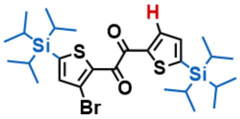

Mechanoresponsive Turn-on RTP

Chem. Commun. **2020**, *56*, 6810–6813.

Properteis in solution

✓ PL spectrum

☐ PLQY & lifetime not reported

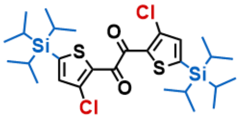

RTP-to-RTP Mechanochromism
with a Modulated Sensitivity

Front. Chem. **2022**, *9*, 812593.

Properteis in solution

☐ Not reported
(manuscript in preparation)

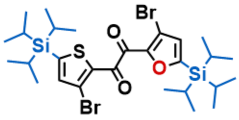

RTP in a Supercooled Liquid State
Chem. Sci. **2021**, *12*, 14363–14368.

Photoinduced Crystal Melting & RTP Photoresponse
Chem. Sci. **2023**, *14*, 5302–5308.

Properteis in solution

✓ PL spectrum, PLQY, lifetime

☐ ISC not evaluated

Figure S3. RTP properties of heteroaromatic 1,2-diketones reported in previous works.

The UV-vis absorption and steady-state PL spectra, Φ_p, and τ_p of other compounds were evaluated in a similar manner (Figures S4–S9 and Table S2). Because of low efficiency, Φ_p and τ_p of **2a**, **2b**, **3a**, and **3b** were evaluated only under Ar and we did not check if k_p changed in air and under Ar.

Table S2. Summary of the Photophysical Properties in Solution[a]

		c /μM	FWHM /nm	Φ_p[b] (%)	τ_p[c] /μs	k_p[d] /s^{-1}	k_{nr}[d] /s^{-1}
**1a**	Ar	10	29	38.2	72.7	5,300	8,500
**1a**	air	10	29	2.6	4.83	5,400	200,000
**1b**	Ar	4.4	32	24.6	49.3	5,000	15,000
**1b**	air	4.4	32	2.5	5.05	5,000	190,000
**1c**	Ar	10	32	17.0	37.7	4,500	22,000
**1c**	air	10	32	2.1	4.85	4,300	200,000
**2a**	Ar	2600	105	0.08	10.6	75	94,000
**2b**	Ar	500	106	0.22	26.2	84	38,000
**3a**	Ar	100	25	0.20	15.7	130	64,000
**3b**	Ar	5.0	26	1.2	37.5	320	26,000

[a] Acquired in cyclohexane at RT. λ_{ex} = 368 nm for all measurements. [b] Determined relative to quinine sulfate. [c] Determined by single exponential fit to the decay. [d] Derived from Φ_p and τ_p according to the formulas $k_p = \Phi_p/\tau_p$ and $k_{nr} = (1 - \Phi_p)/\tau_p$.

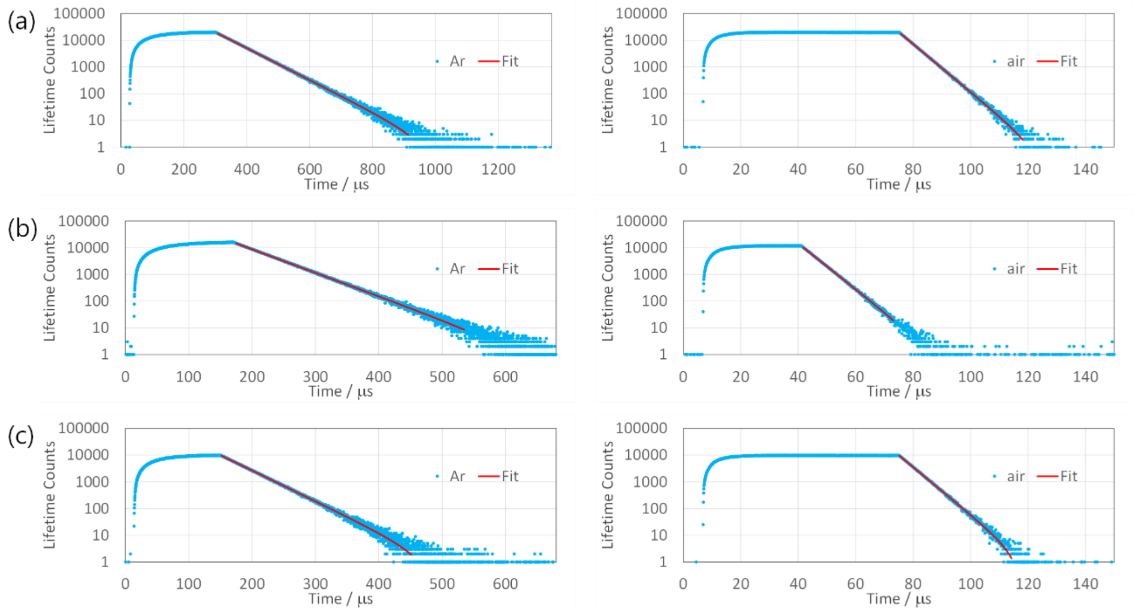

Figure S4. PL decay curves of **1a**–**1c** (from top to bottom) in cyclohexane at RT (λ_{ex} = 368 nm). Left, under Ar; right, in air. The red lines are the single-exponential fit to the curves. PL intensities were recorded at 560, 570, and 568 nm for **1a**, **1b**, and **1c**, respectively.

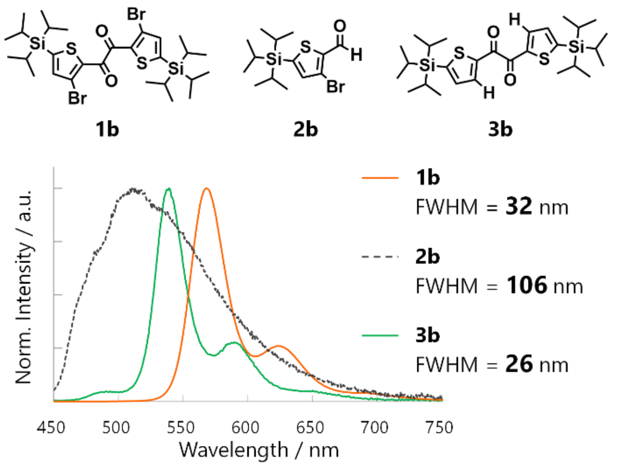

Figure S5. Chemical structures and steady-state PL spectra of **1b**, **2b**, and **3b** in cyclohexane (4.4×10^{-6} M, 5.0×10^{-4} M, and 5.0×10^{-6} M, respectively) excited at 368 nm under Ar, and corresponding full-width-at-half-maxima (FWHM).

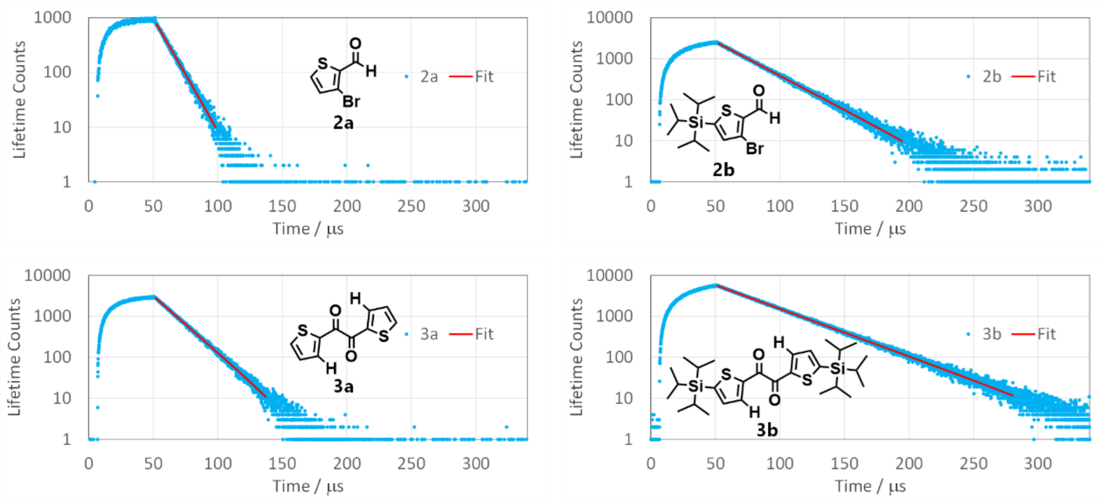

Figure S6. PL decay curves of **2a**, **2b**, **3a**, and **3b** in cyclohexane at RT under Ar (λ_{ex} = 368 nm). PL intensities were recorded at 500, 510, 530, and 538 nm, respectively. The red lines are the single-exponential fit to the curves.

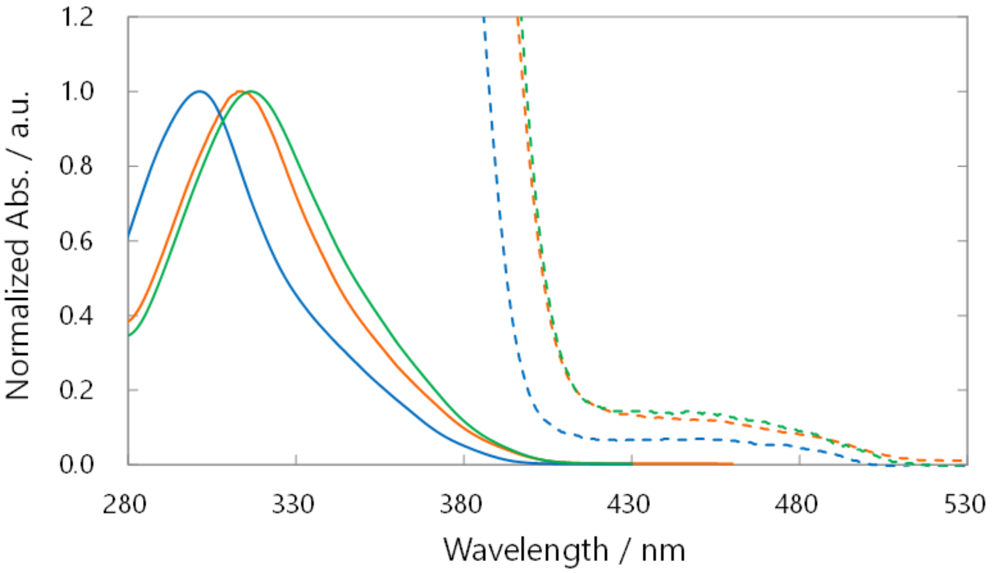

Figure S7. Normalized UV-vis absorption spectra of **1a** (blue, 1.0 × 10^{-4} M), **1b** (green, 4.4 × 10^{-5} M), and **1c** (orange, 1.0 × 10^{-4} M) in cyclohexane at RT. The broken lines are enlarged 40-times.

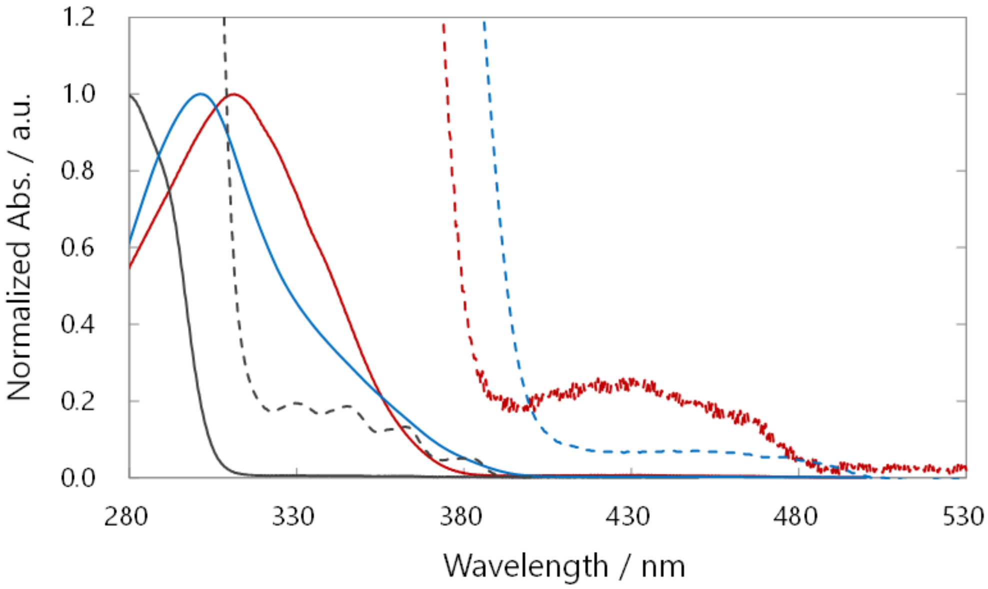

Figure S8. Normalized UV-vis absorption spectra of **1a** (blue, 1.0 × 10^{-4} M), **2a** (black, 1.3 × 10^{-4} and 2.6 × 10^{-3} M for solid and broken lines, respectively), and **3a** (red, 1.0 × 10^{-4} M) in cyclohexane at RT. The broken lines are enlarged 40-times.

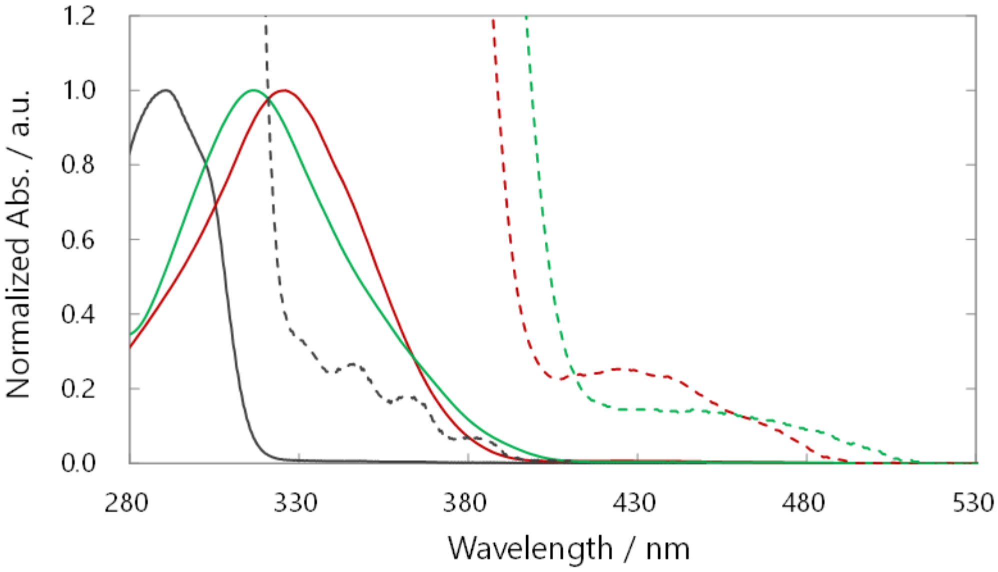

Figure S9. Normalized UV-vis absorption spectra of **1b** (green, 4.4 × 10^{-5} M), **2b** (black, 5.0 × 10^{-5} and 5.0 × 10^{-4} M for solid and broken lines, respectively), and **3b** (red, 1.0 × 10^{-4} M) in cyclohexane at RT. The broken lines are enlarged 40-times.

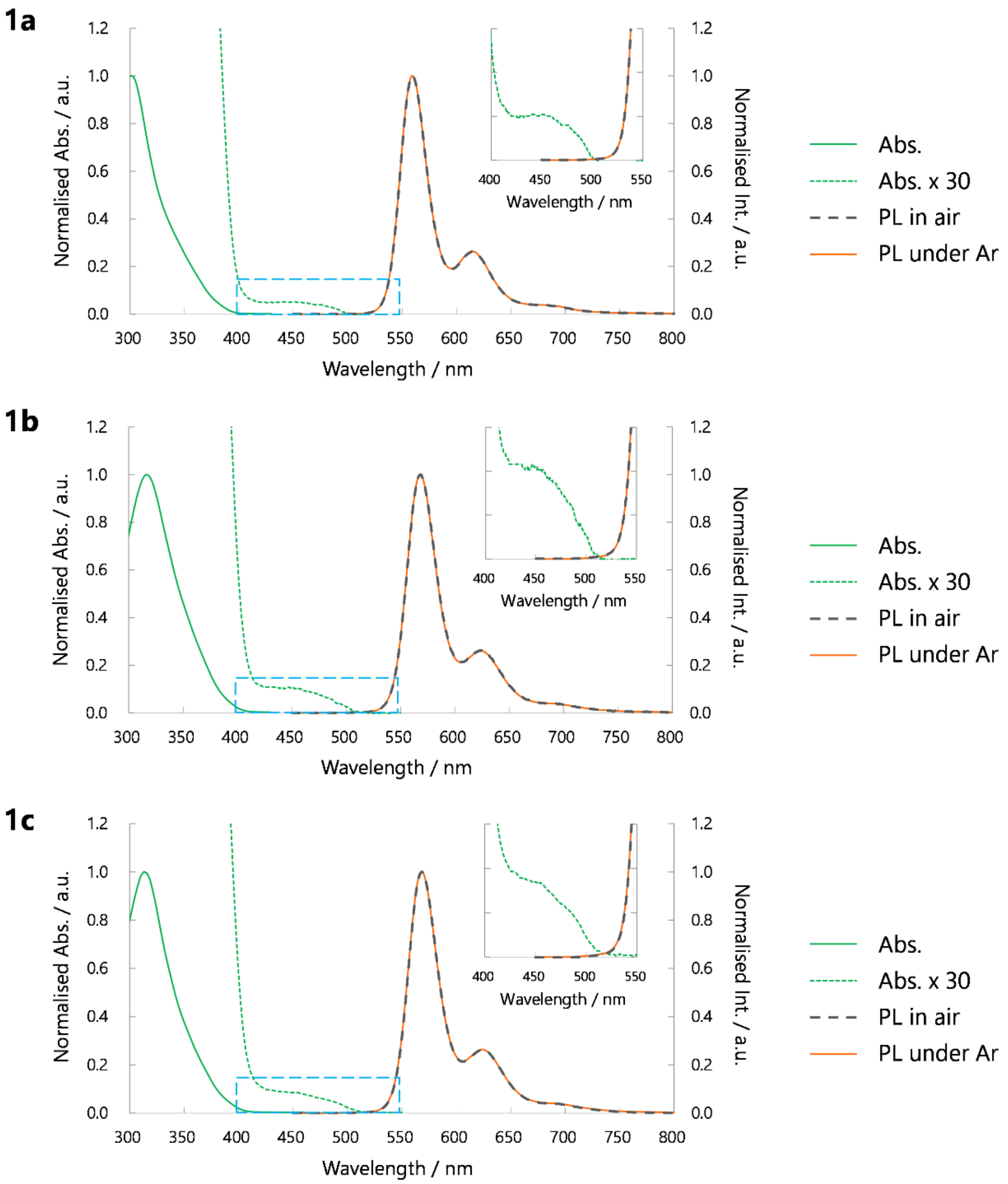

Figure S10. UV-vis absorption and steady-state PL spectra in air and under Ar for **1a**, **1b**, and **1c** in cyclohexane. There is no discernible fluorescence emission in the steady-state PL spectra between the onset of absorption and phosphorescence. Moreover, the normalised PL spectrum in air matches that under Ar from the onset of the PL spectra.

Rising time scale of phosphorescence emission

The time-resolved photoluminescence (TRPL) measurements were performed using a streak camera system (Hamamatsu C4780, time resolution <100 ps) synchronized with a Ti:sapphire regenerative amplifier (Spectra-Physics, Spitfire Ace, pulse duration: 120 fs, repetition rate: 1 kHz, pulse energy: 4 mJ/pulse, wavelength: 800 nm).[14] The samples were excited at 350 nm using the fourth harmonic of the output of optical parametric amplifier pumped by the fundamental pulse from an amplifier.

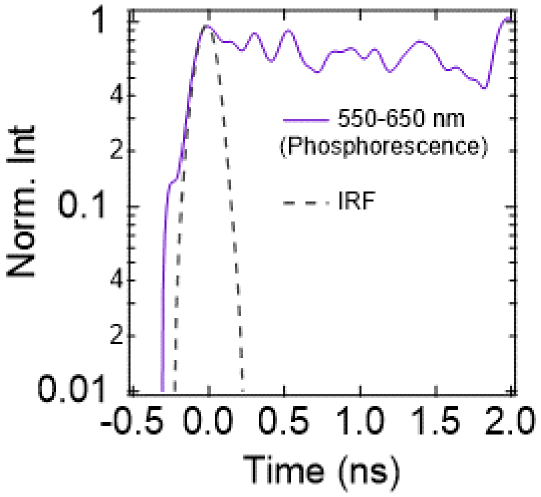

Figure S11. TRPL of **1a** in cyclohexane at the phosphorescence region. The PL intensity rose in instrumental response function (<100 ps).

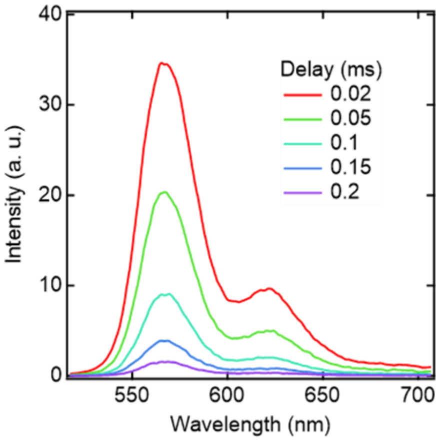

Figure S12. TRPL spectra of **1a** in cyclohexane.

Transient absorption spectroscopy

The femtosecond transient absorption (fsTA) and nanosecond transient absorption (nsTA) measurements were performed utilizing the pump-probe method.[15] A Ti:sapphire regenerative amplifier system (Spectra-Physics, Spitfire Ace, pulse duration: 120 fs, repetition rate: 1 kHz, pulse energy: 4 mJ/pulse, wavelength: 800 nm) was employed as the light source.

For fsTA, the amplifier output was divided into two pulses for the pump and probe purposes. The samples were pumped with the fourth harmonic of the output of optical parametric amplifier pumped by the fundamental pulse from an amplifier. To generate the broadband probe pulse (450–750 nm), a 3 mm-thick sapphire crystal was employed. A PC-controlled mechanical delay stage was utilized to adjust the time delay between the pump and probe pulses. The probe pulse that passed through the sample films was dispersed using a polychromator (JASCO, CT-10, 300 grooves/500 nm), and the resulting spectra were recorded by a multichannel detection system equipped with a CMOS sensor (UNISOKU, USP-PSMM-NP). The collected data was analyzed using a homemade Python-based program.

For nsTA, the broadband probe pulse was generated by the same setup as fsTA. The sample was pumped by the third harmonic of Nd:YAG laser (EKSPLA, NT242, pulse duration: 6 ns, repetition rate: 1 kHz, wavelength: 355 nm).

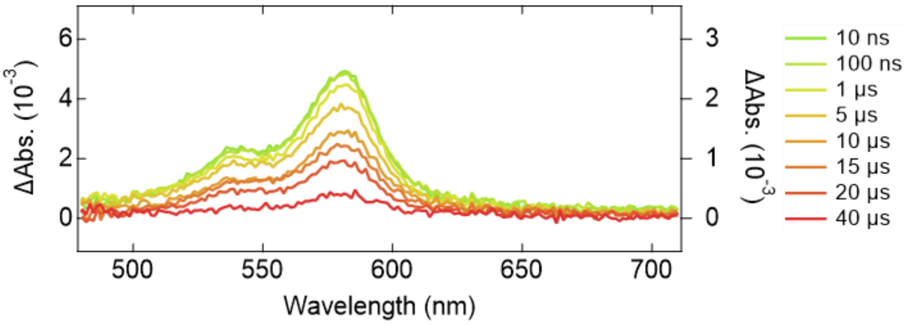

Figure S13. nsTA spectra of **1a**. No spectral change was observed in the timescale.

Global Analysis

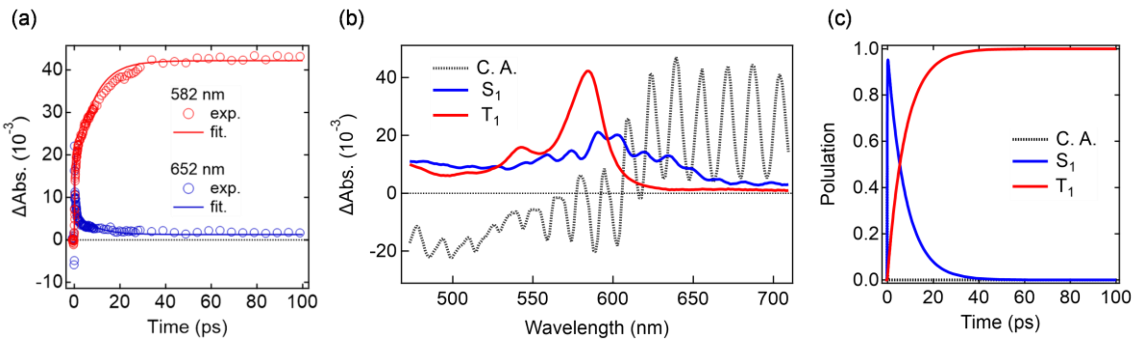

Figure S14. Complete global analysis of the TAS of **1a** in cyclohexane solution. (a) Selected comparison of the experimentally observed time courses of the TA and the result of the global fitting assuming sequential model with three states. (b) Evolution associated spectra. The first component with structureless interference pattern is assigned to the coherent artefact, the second and the third components are assignable to the S_1 and T_1 state. (c) Temporal profiles of three species (coherent artefact: C. A., S_1 and T_1).

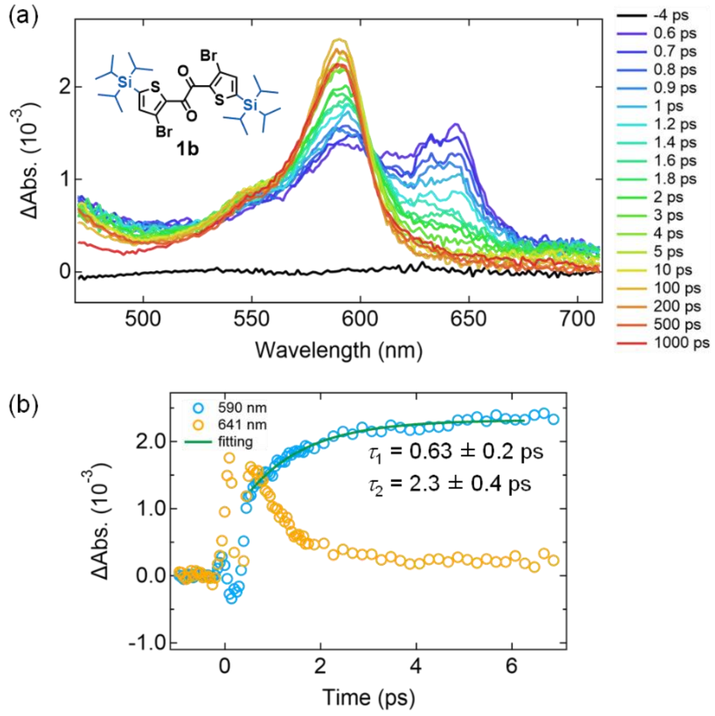

Figure S15. (a) TAS spectra obtained from a cyclohexane solution of **1b** upon excitation at 400 nm. (b) Temporal evolution of the TA signal at the selected wavelength: 590 nm (blue) and 641 nm (orange). The fitting curve of the time trace at 590 nm using double exponential function is also shown.

Time-resolved infrared spectroscopy (TRIR)

TRIR spectra were acquired by a purpose-built system based on the pump-probe method, using a femtosecond Ti:sapphire chirped pulse amplifier (Spectra-Physics, Spitfire Ace, pulse duration: 120 fs, repetition rate: 1 kHz, pulse energy: 4 mJ/pulse, wavelength: 800 nm). The pump pulse (wavelength: 267 nm, fluence at sample position = 7 mJ/cm^2) was generated using the third harmonic generation of part of the amplifier output. The tunable probe pulse (wavenumber range = 1000−4000 cm^{-1}, bandwidth = 150 cm^{-1}) was obtained by an optical parametric amplifier equipped with a crystal for difference frequency generation (Lighconversion, TOPAS-Prime). The pump and probe pulses were irradiated on an infrared flow cell with BaF_2 windows (optical path length: 0.1). The polarizations of the pump and probe pulses on the flow cell were set to the magic angle using half-wave plates for visible and mid-infrared wavelengths to avoid the effect of rotational relaxation. The spectrum of each probe pulse after passing through the flow cell was recorded by a mercury−cadmium−tellurium (MCT) infrared linear array system equipped with a 19 cm polychromator (Infrared Systems Development FpAS-64166-D). TR- IR spectra were obtained by averaging the difference between those with and without the pump pulse using an optical chopper synchronized with the pump pulse. All measurements were carried out at room temperature.

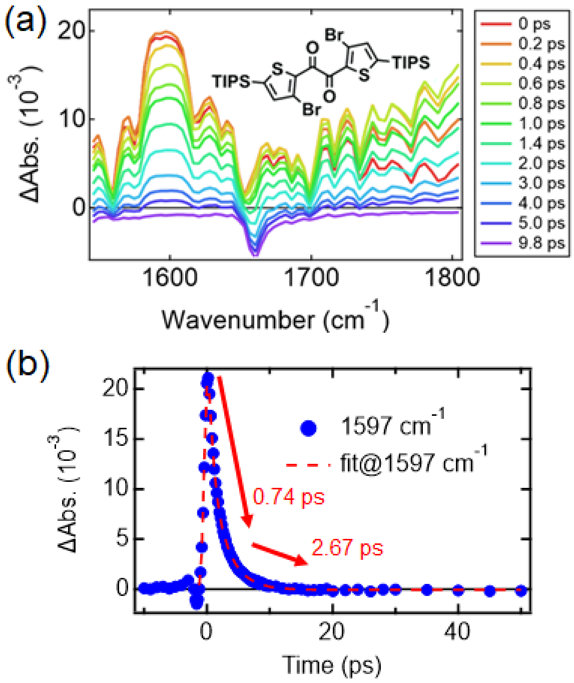

Figure S16. (a) TRIR spectra obtained from a cyclohexane solution of **1b** upon excitation at 267 nm. (b) Temporal evolution of the TRIR signal at 1597 cm^{-1}. The fitting curve of the time trace at 1597 cm^{-1} is also shown.

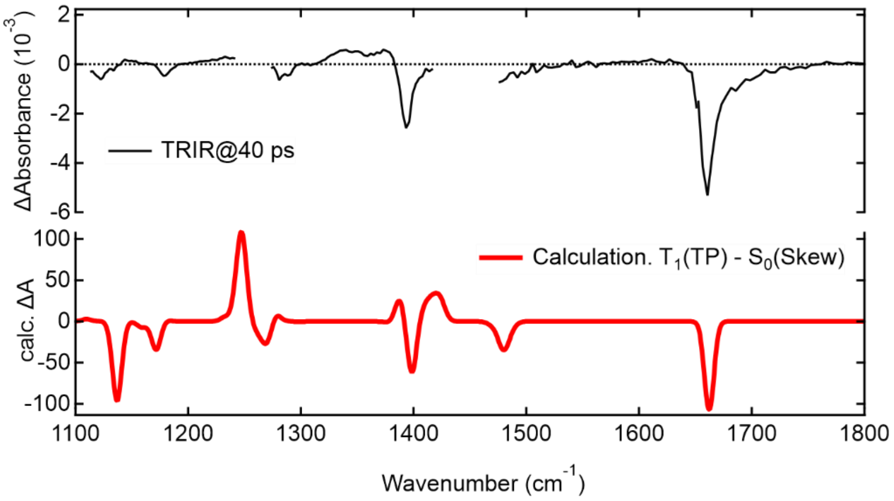

Figure S17. The comparison of the experimentally obtained TRIR spectra of **1b** (black) and quantum chemical calculations of **1b** assuming the TP conformer in the T_1 state and a skew conformer in the S_0 state (red). Because of the strong IR absorption of the solvent (cyclohaxane) causing no transmission of the probe IR pulse, the TRIR data could not be detected at around ~1250 cm^{-1} and ~1450 cm^{-1}. Note that the TP conformer was 6.9 kcal mol^{-1} more stable than the skew conformer at the T_1-minima, while was 0.79 kcal mol^{-1} less stable at the S_0-minima. The calculations were based on density functional theory (B3LYP-D3/6-311G(d)). The scaling factor was set to 0.97.

Franck–Condon analysis of the emission spectra.

The emission spectra of **1a**, **1b**, **2a**, and **2b** were fitted using a Franck–Condon expression assuming one vibronic mode using the following equation:[16]

$$I(\tilde{\nu}) = \sum_{\nu_m=0}^{\infty} \left(\frac{E_{00} - \nu_m \hbar\omega_m}{E_{00}}\right)^4 \left(\frac{S_m^{\nu_m}}{\nu_m!}\right) \exp\left[-4\ln 2\left(\frac{\tilde{\nu} - E_{00} + \nu_m \hbar\omega_m}{\Delta\tilde{\nu}_{1/2}}\right)^2\right] \quad \text{... (S1)}$$

where $I(\tilde{\nu})$ is the emission intensity at energy, E_{00} is the energy of the zero-zero transition, ν_m is the quantum number of the vibrational mode, $\hbar\omega_m$ is the quantum spacing for the averaged vibrational modes governing nonradiative decay, S_m is the Huang–Rhys factor,[17] and $\Delta\tilde{\nu}_{1/2}$ is the full-width-at-half-maximum (FWHM) for an individual vibronic line. The summation was carried out over three or six vibrational levels. The photon numbers for the emission spectra were corrected using a wavenumber scale by the equation: $I(\tilde{\nu}) = I(\lambda) \times \lambda^2$.[18] The fitting results for **1b** and **2b** are shown in Figure S18. The optimized parameters are shown in Table S3. To evaluate color purity of the spectra that accompany vibronic bands, we have calculated the area ratio of the largest vibronic band to the whole spectrum, and included in the table.

According to the spacing, the normal mode involved in the transition was assigned to the stretching vibration of the 1,2-dicarbonyl moiety (Figure S19). This mode hardly involves triisopropylsilyl substituents, resulting in the similar FWHM for **1a** and **1b**.

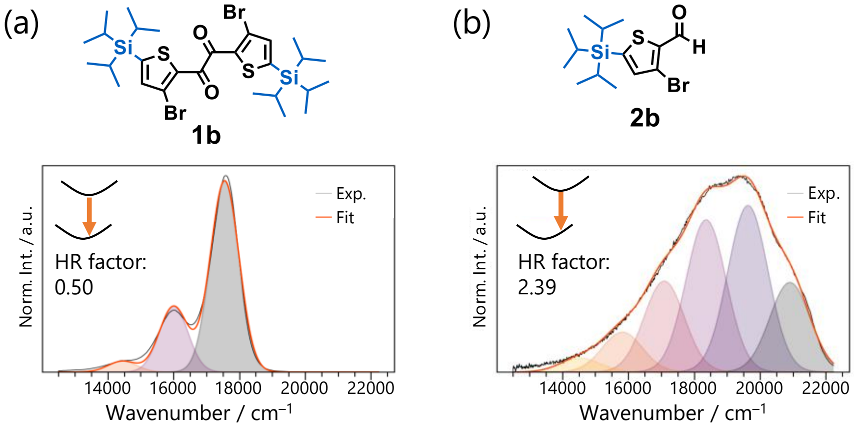

Figure S18. Franck–Condon analyses of the PL spectra of diketone **1b** and aldehyde **2b** in cyclohexane.

Table S3. The Optimized Parameters of Emission Spectra Fitting and Area Ratio.

	HR factor	Spacing /cm^{-1}	E_{00} /cm^{-1}	FWHM /cm^{-1}	Area ratio[a]
**1a**	0.51	1568.2	17816.9	985.6	71
**2a**	2.22	1432.7	21342.3	1702.4	30
**1b**	0.50	1552.3	17548.8	1023.9	72
**2b**	2.39	1271.4	20899.0	1439.3	30

[a]The ratio of the largest vibronic band to the whole spectrum.

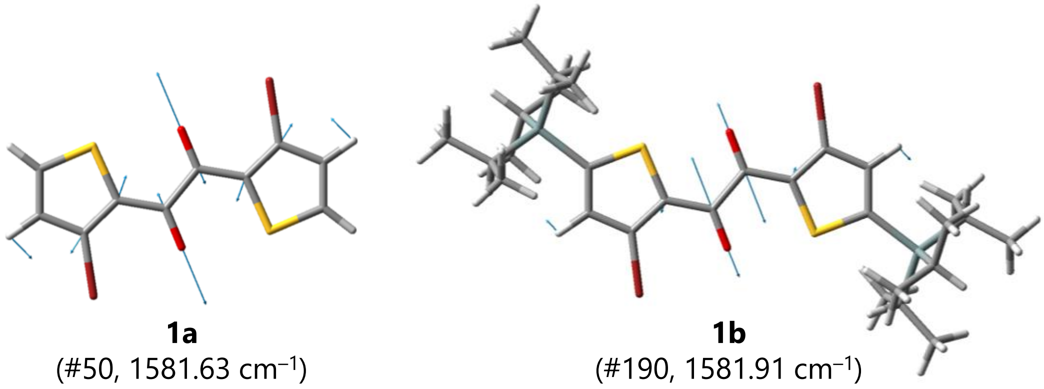

Figure S19. Stretching vibrations of **1a** and **1b** vibronically coupled to phosphorescence. Obtained by S_0-state frequency calculations at T_1-minimum geometries.

Evaluating steady-state PL spectrum, Φ_p, and τ_p of the diketone@polymer films

Typically, **1a** (4.7 mg) and PMMA (Aldrich #182230, 90.0 mg) were dissolved into $CHCl_3$ (3 mL, spectral-grade). The solution (100 μL) was dropped onto a 10 × 10 mm^2 quartz substrate and spin-coated at 100 rpm for 10 sec and then at 1000 rpm for 200 sec. The film was dried under vacuum at RT overnight. Source of other polymers are: ZEONEX® 480R, Zeon co.; polystyrene (PS), Aldrich #182427; polyvinylformal (PVF), TCI P0641; poly(D,L-lactide) (PLA), Aldrich #719951; polyvinylpyrrolidone (PVP), TCI P0472.The PL spectra were measured using a JASCO FP-8200 spectrofluorometer with a L37 sharp cut filter (HOYA, long pass, >370 nm). For PMMA and zeonex films, a U340 band pass filter (HOYA) was used. Φ_p of the films were determined using a Hamamatsu photonics C11347-01 or C9920-02 spectrometer with an integrating sphere.

The PL decay curves were measured using a HORIBA DeltaFlex multichannel scaling system using DeltaDiode for excitation (368 nm) for **1c**@PMMA or a HORIBA Fluorolog3-211 spectrometer for others.

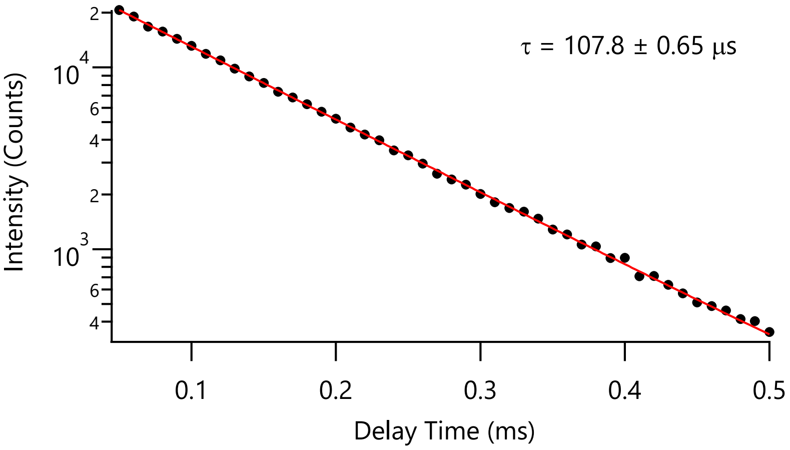

Figure S20. PL decay curve of PMMA film doped with 5 wt% of **1a** at RT in air. The red line denotes the single-exponential fit to the curve. The PL intensity at 550 nm was recorded (λ_{ex} = 365 nm).

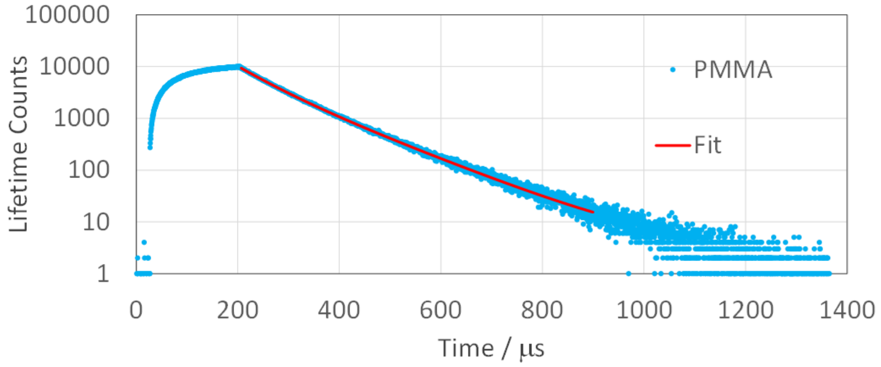

Figure S21. PL decay curve of PMMA film doped with 5 wt% of **1c** at RT in air. The red line denotes the double-exponential fit to the curve. The PL intensity at 570 nm was recorded (λ_{ex} = 368 nm).

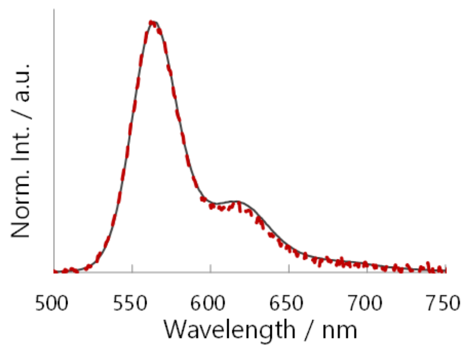

Figure S22. Steady-state PL spectra of PMMA film doped with 5 wt% of **1c** at RT in air (black) and in water (red). λ_{ex} = 368 nm.

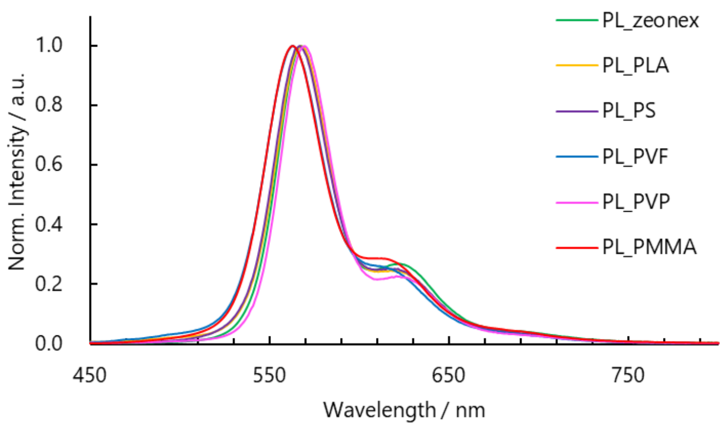

Figure S23. Steady-state PL spectra of various polymer film doped with 5 wt% of **1b** at RT in air (λ_{ex} = 300 nm).

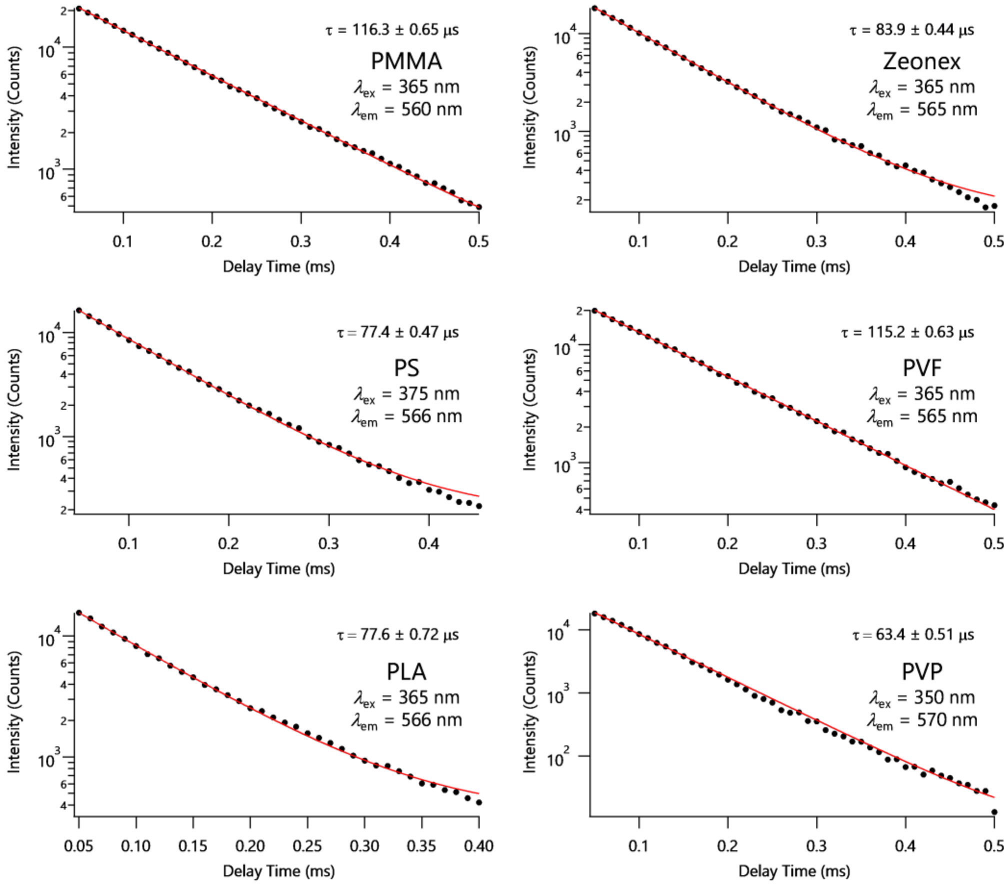

Figure S24. PL decay curves of various polymer films doped with 5 wt% of **1b** at RT in air. The red lines denote the single-exponential fit to the curves.

Table S4. Summary of Photophysical Properties of the Polymer Films

		FWHM /nm	λ_{em} [a] /nm	Φ_p [b] (%)	τ_p [c] /μs	σ [c] /μs
**1b**	c-Hex (Air)	32	570	2.5[d]	5.05	±0.0084
	c-Hex (Ar)	32	570	24.6[d]	49.3	±0.0288
	PMMA	36	563	48 (45)	116.3	±0.65
	Zeonex	33	567	24	83.9	±0.44
	PS	35	567	19	77.4	±0.47
	PVF	38	563	38	115.2	±0.63
	PLA	37	568	18	77.6	±0.72
	PVP	34	570	18	63.4	±0.51
**1a**	PMMA	35	555	54 (44)	107.8	±0.65
**1c**	PMMA	36	563	40 (39)	87.8[e]	±1.2[e]

[a]Emission maxima. [b]Absolute quantum yields measured using an integrating sphere. Averages of three to five films are given in parentheses. [c]Phosphorescence lifetimes and their standard deviations obtained by a single exponential fit to the decay curves using the igor software. [d]Relative quantum yields. [e]Area-weighted averaged lifetime and its standard deviation obtained by a double exponential fit to the decay curves using a HORIBA EzTime software.

Procedure for evaluating steady-state PL spectrum, Φ_p, and τ_p of 1c crystal

To a $CHCl_3$ solution of **1c** was added large excess of MeOH, and the solution was cooled in an ice bath and kept for 3 h to obtain the crystal of **1c**. The steady-state PL spectrum and Φ_p of **1c** crystal at RT in air was obtained using a Hamamatsu photonics C9920-02 spectrometer with an integrating sphere (λ_{ex} = 368 nm). The Φ_p was determined to be 50.5% as an average of four measurements. PL decay curve at 570 nm at RT in air was acquired using a HORIBA DeltaFlex multichannel scaling system using DeltaDiode for excitation (368 nm). τ_p was obtained as 79.2±0.16 μs from a single-exponential fit to the decay curve using a HORIBA EzTime software.

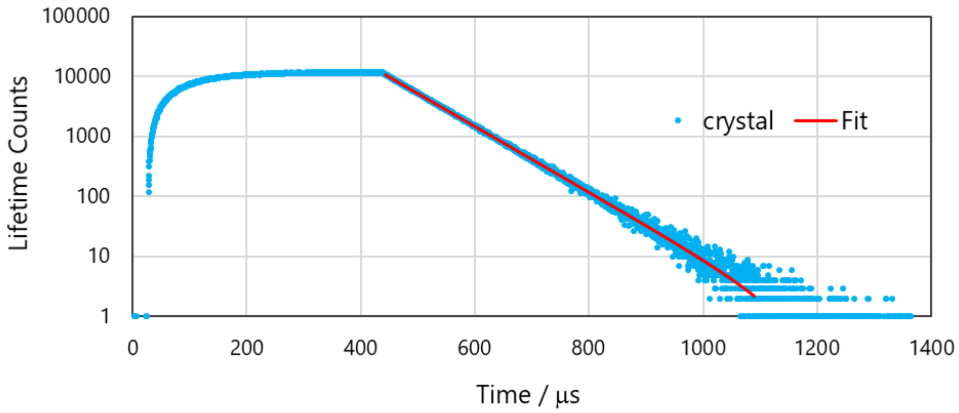

Figure S25. PL decay curve of **1c** crystal at RT in air (λ_{ex} = 368 nm). The red line is the single-exponential fit to the curve. PL intensity was recorded at 570 nm.

5. Theoretical Calculations

All geometry optimizations and frequency calculations at S_0 and T_1 states were performed using density functional theory (DFT) method at the (u)B3LYP-D3/6-311G(d) level of theory as implemented in Gaussian 16 program package.[19] Likewise, all geometry optimizations and frequency calculations at S_1 states were performed using time-dependent DFT (TDDFT) method at the B3LYP-D3/6-311G(d) level of theory. None of the optimized structures had imaginary frequencies.

Single-point calculations of energy levels, transition dipole moments between S_n and S_0 states $\boldsymbol{\mu}_{Sn\text{–}S0}$, and spin–orbit coupling matrix elements between S_n and T_1 states $\langle S_n|\mathbf{H}_{SO}|T_1\rangle$ or S_1 and T_n states $\langle S_1|\mathbf{H}_{SO}|T_n\rangle$ were conducted using the Tamm–Dancoff approximation (TDA) of time-dependent DFT (TDDFT) method at the uCAM-B3LYP-D3BJ/6-311G(d) level of theory as implemented in ORCA program package.[20] The use of coulomb-attenuated functional coupled with TDA was reported as a powerful approach to calculate triplet excitation energies.[21] For natural transition orbital (NTO) analysis, single-point TDDFT calculations were also performed using Gaussian 16 program.

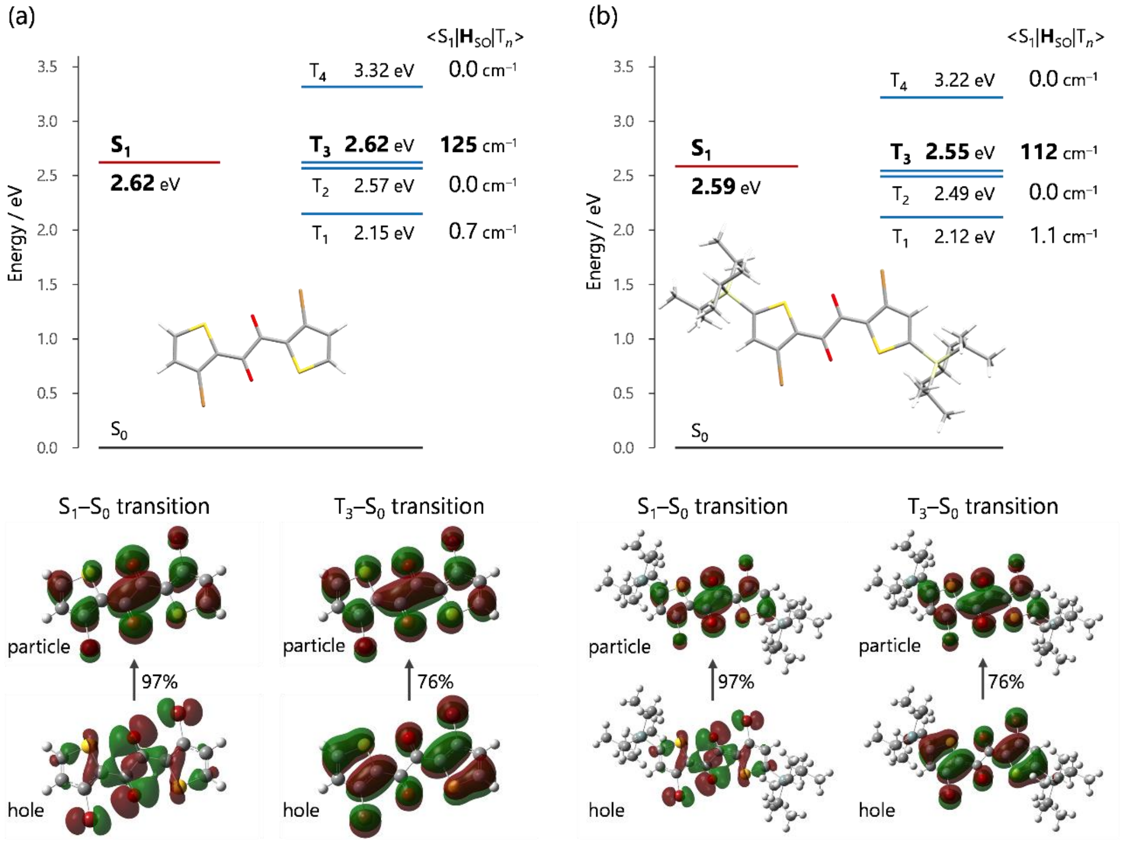

Figure S26. Energy diagrams, $\langle S_1|\mathbf{H}_{SO}|T_n\rangle$, and selected NTOs (for the S_1 and T_3 states) of **1a** (a) and **1b** (b) at the S_1-optimized TP geometry.

Table S5. Factors of Intensity Borrowing Scheme for k_p Calculated at the T_1-Optimized Geometry

		$\Delta E_{S_n\text{–}T_1}$ / eV	$\langle S_n\|\mathbf{H}_{SO}\|T_1\rangle$ / cm^{-1}	mixing coeff. (%)	$\|\mu_{S_n\text{–}S_0}\|$ / Debye	$\|m_p^n\| \times 10^3$ / Debye			$\Delta E_{S_n\text{–}T_1}$ / eV	$\langle S_n\|\mathbf{H}_{SO}\|T_1\rangle$ / cm^{-1}	mixing coeff. (%)	$\|\mu_{S_n\text{–}S_0}\|$ / Debye	$\|m_p^n\| \times 10^3$ / Debye
**1a**	S_1	0.48	1	0.02	0.01	0.0	**1b**	S_1	0.47	1	0.02	0.05	0.0
	S_2	1.67	0	0.00	0.00	0.0		S_2	1.66	149	1.11	5.38	59.8
	S_3	1.74	167	1.19	4.68	55.7		S_3	1.68	0	0.00	0.00	0.0
	S_4	1.88	0	0.00	0.00	0.0		S_4	1.82	0	0.00	0.00	0.0
	S_5	2.04	0	0.00	0.00	0.0		S_5	1.94	0	0.00	0.00	0.0
	S_6	2.23	12	0.07	4.68	3.2		S_6	2.08	16	0.10	5.93	5.8
**2a**	S_1	1.25	51	0.51	0.08	0.4	**2b**	S_1	1.29	49	0.47	0.08	0.4
	S_2	2.32	0	0.00	4.36	0.0		S_2	2.15	1	0.00	4.36	0.2
	S_3	2.64	0	0.00	2.91	0.0		S_3	2.54	1	0.00	2.91	0.1
	S_4	3.15	73	0.29	0.23	0.7		S_4	3.24	66	0.25	0.23	0.5
	S_5	3.49	247	0.88	0.22	2.0		S_5	3.24	63	0.24	0.22	1.3
	S_6	3.95	49	0.16	0.03	0.0		S_6	3.39	75	0.27	0.03	3.4
**3a**	S_1	0.54	0	0.01	0.08	0.0	**3b**	S_1	0.55	0	0.01	0.08	0.0
	S_2	1.82	6	0.04	0.50	0.2		S_2	1.80	12	0.08	4.10	3.3
	S_3	1.96	23	0.15	5.16	7.6		S_3	1.80	17	0.12	6.03	7.0
	S_4	1.98	0	0.00	0.15	0.0		S_4	1.88	0	0.00	0.15	0.0
	S_5	2.16	3	0.01	0.47	0.1		S_5	2.08	3	0.02	0.43	0.1
	S_6	2.22	12	0.07	3.02	2.1		S_6	2.12	16	0.10	2.46	2.4

The phosphorescence rate constants k_p were numerically calculated with factors in Table S5 following Equations S1, 2, and 3 (for n = 1–6; see main text for Equations 2–3):

$$k_p = \frac{64\pi^4}{3h^4c^3}\left(\Delta E_{T_1-S_0}\right)^3 |\boldsymbol{M}_p|^2 \text{ ... (S1)}$$

where h is Plank constant and c is the speed of light.

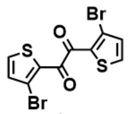

1a

k_p (calc.) = **5400** s^{-1}
k_p (exp.) = **5300** s^{-1}

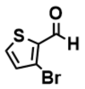

2a

k_p (calc.) = **9** s^{-1}
k_p (exp.) = **75** s^{-1}

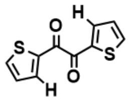

3a

k_p (calc.) = **71** s^{-1}
k_p (exp.) = **130** s^{-1}

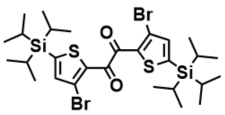

1b

k_p (calc.) = **4900** s^{-1}
k_p (exp.) = **5000** s^{-1}

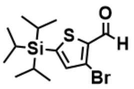

2b

k_p (calc.) = **18** s^{-1}
k_p (exp.) = **84** s^{-1}

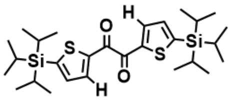

3b

k_p (calc.) = **52** s^{-1}
k_p (exp.) = **320** s^{-1}

Figure S27. Calculated and experimental k_p values.

Single-point calculations of aldehydes **2a** and **2b** and Br-free diketones **3a** and **3b** were performed on the s-*cis* conformer, because those were more stable than the s-*trans* conformers (Figure S28 and Figure S29). The elongation ratio (Figure 7c) was obtained as the ratio of bond length in the S_0- and T_1-minimum geometries (TP conformer for **1a** and s-*cis* conformer for **2a**).

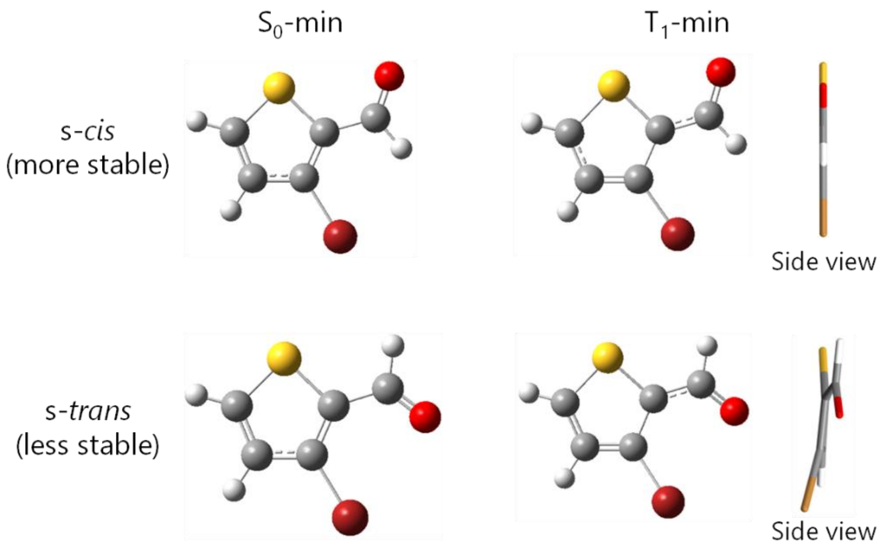

Figure S28. S_0- and T_1-optimized s-*cis* and s-*trans* geometries of **2a**. s-*cis* conformers are 3.19 and 3.43 kcal mol^{-1} more stable than s-*trans* conformers at S_0 and T_1 states, respectively. The s-*trans* geometry at T_1 minimum is significantly bent likely due to the steric repulsion between the oxygen and bromine atoms.

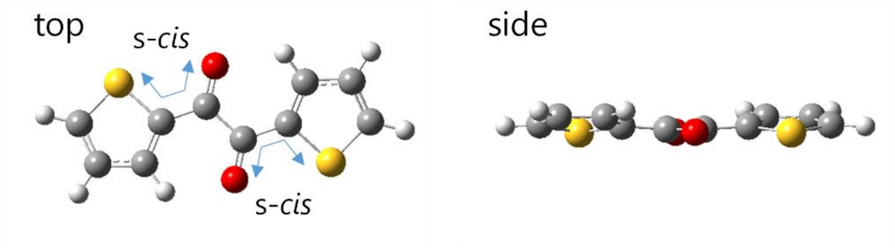

Figure S29. The optimized geometry of the most stable conformer of **3a** at the T_1 state.

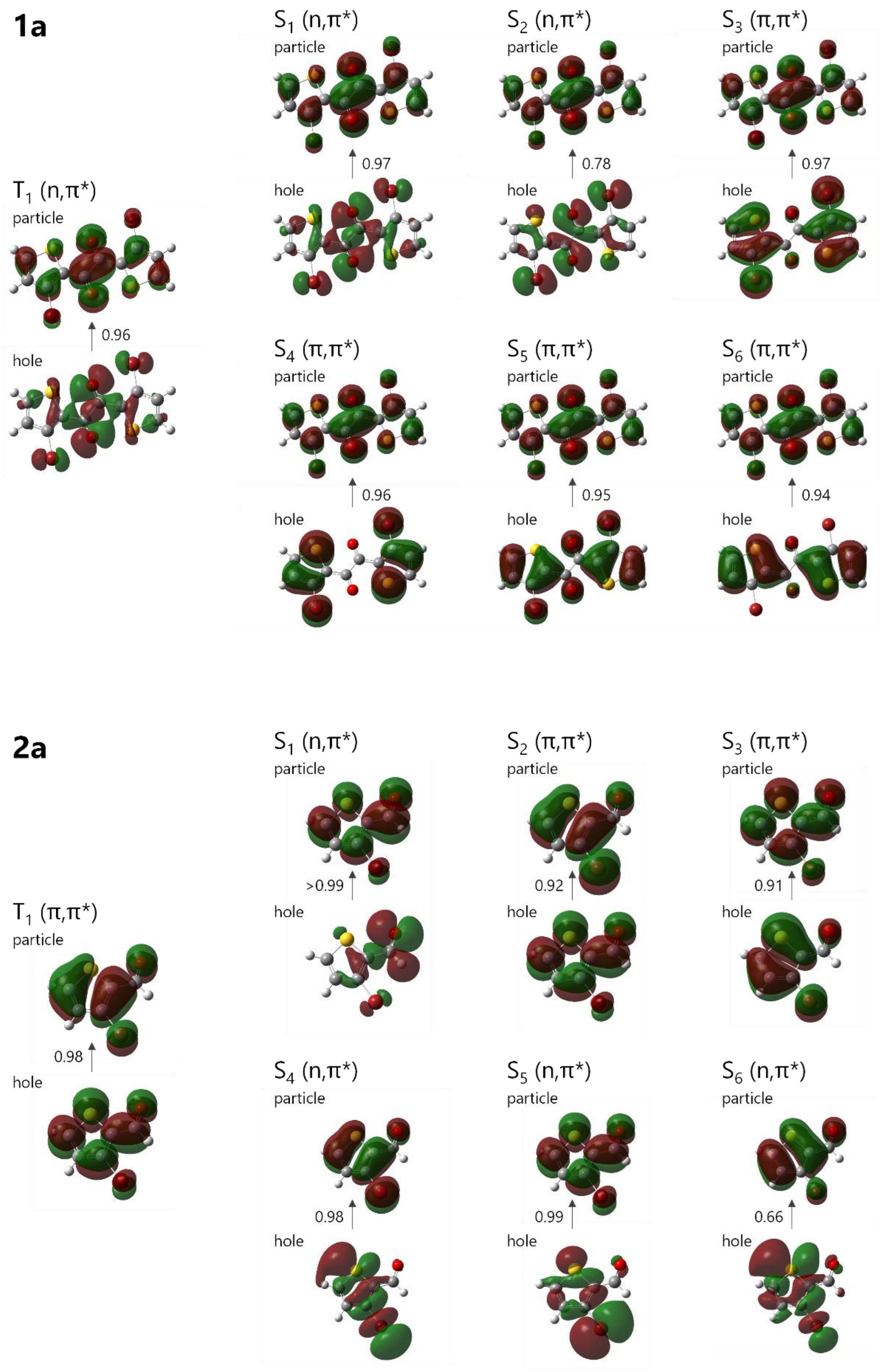

Figure S30. The major NTOs of **1a** and **2a** calculated at TDA/uCAM-B3LYP-D3/6-311G(d) level. Geometry was optimized at the T_1 state.

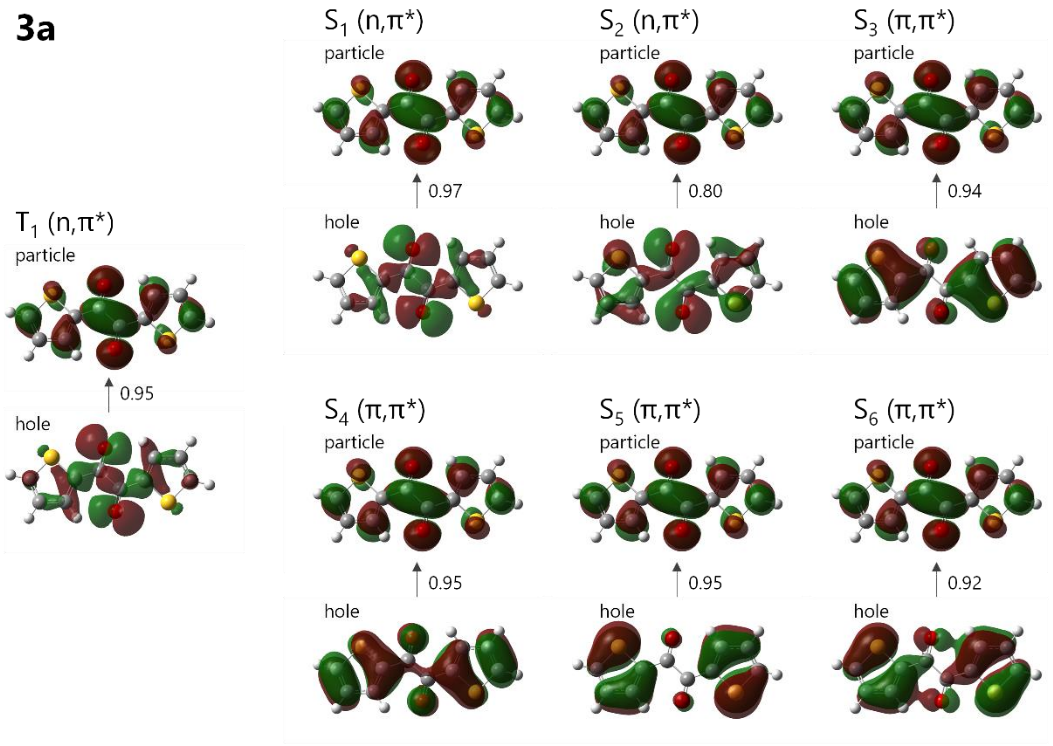

Figure S31. The major NTOs of **3a** calculated at TDA/uCAM-B3LYP-D3/6-311G(d) level. Geometry was optimized at the T_1 state.

6. Single-Crystal X-ray Structure Analysis

Single-crystal of **1c** suitable for the X-ray crystal structure analysis was obtained by cooling $(Me_3Si)_2O$ solution. Data were collected on a Rigaku XtaLAB Synergy-R diffractometer using multilayer mirror monochromated Mo Kα radiation (λ = 0.71075 Å) in the ω scan mode. The crystal was cooled by a stream or cold N_2 gas. Collection, indexing, peak integration, cell refinement, and scaling of the diffraction data were performed using the CrysAlisPro software (Rigaku). The structure was solved by direct methods (SHELXT 2018) and refined by full-matrix least-square refinement on F^2 (SHELXL 2018). The non-hydrogen atoms were refined anisotropically. All hydrogen atoms were placed on the calculated positions and refined using the riding model.

Table S6. Crystallographic Data for 1c (CCDC 2269866)

Empirical formula	$C_{34}H_{56}Br_2O_2S_2Si_2$
FW	776.90
T / K	123
Crystal system	triclinic
Space group	P-1
a / Å	9.4163(4)
b / Å	10.5452(5)
c / Å	10.6006(5)
α / °	76.484(4)
β / °	77.312(4)
γ / °	76.710(4)
V / Å^3	980.61(8)
Z	1
D (calcd) / g·cm^{-3}	1.316
Crystal size/mm^3	0.3 × 0.2 × 0.15
GOF on *F2*	1.024
R1 [$I > 2\sigma(I)$]	0.0254
wR2 (all data)	0.0667
Largest diff. peak/hole / e Å^{-3}	0.40/-0.36

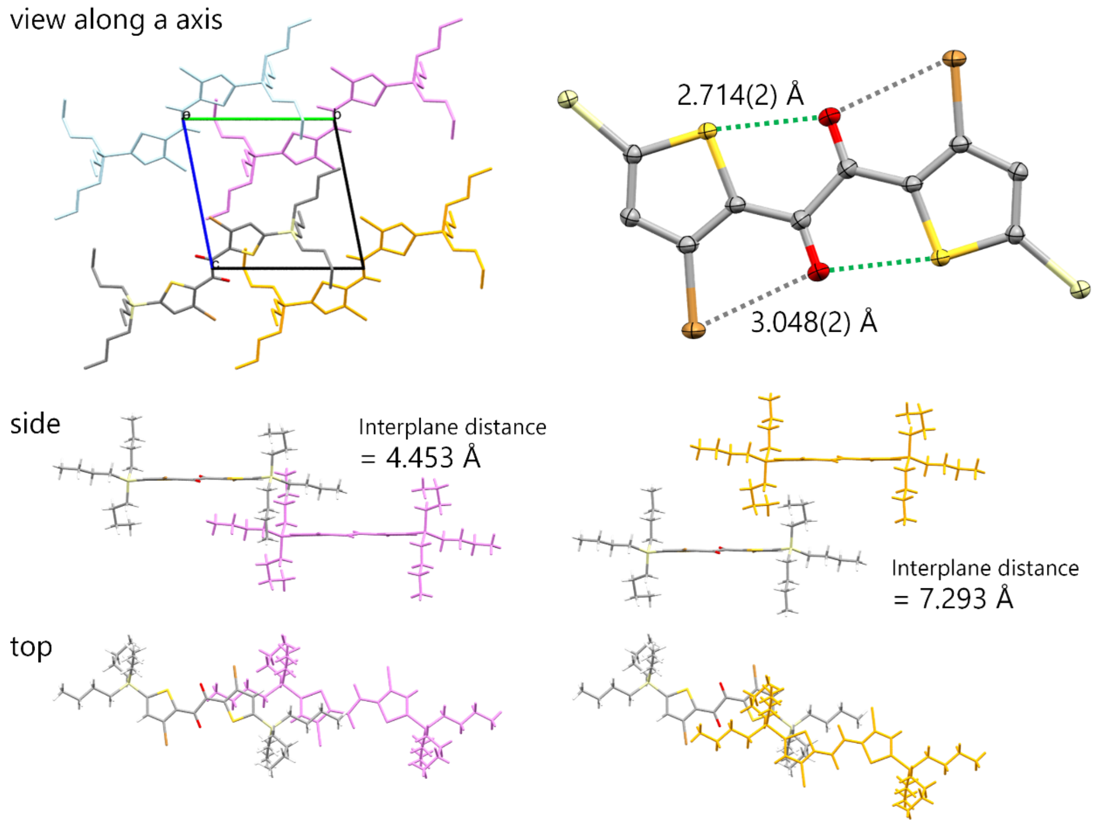

Figure S32. Single-crystal X-ray structure of **1c**. The intramolecular distances between S…O and O…Br are shorter than the sum of the van der Waals radii, which are 3.39 and 3.36 Å for S…O and O…Br, respectively.[22]

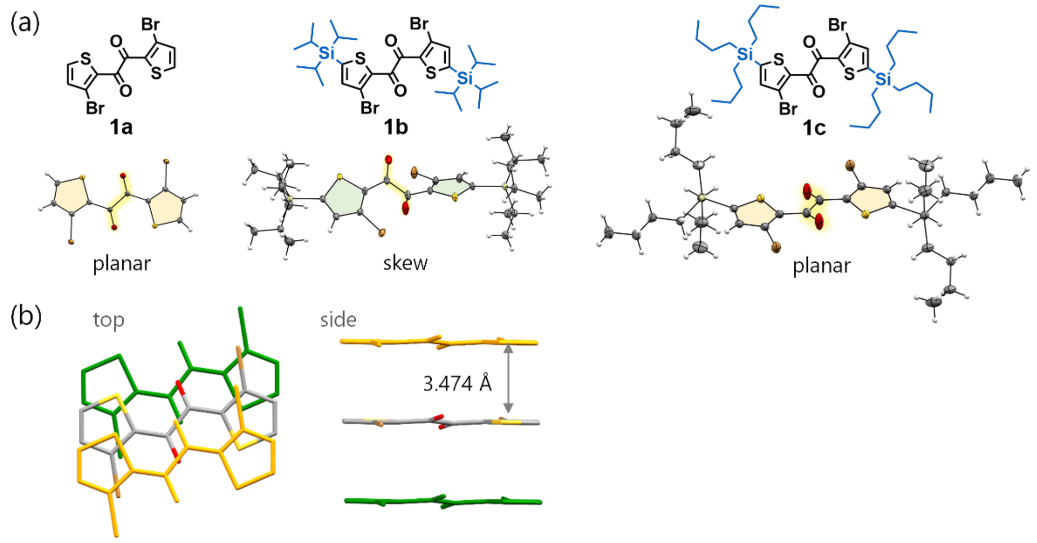

Figure S33. (a) Comparison of chemical and single-crystal X-ray structures of **1a**, **1b**, and **1c**. (b) Crystal packing of **1a**.

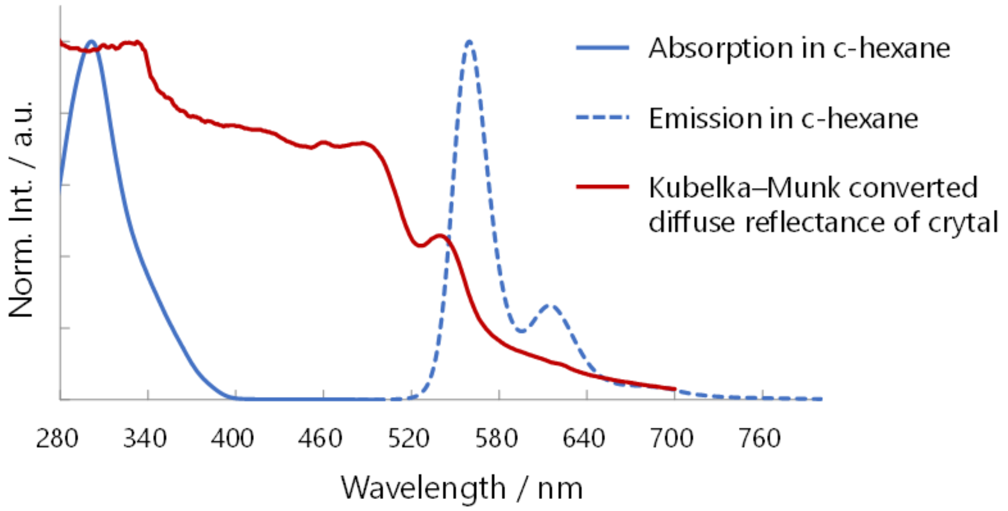

Figure S34. Kubelka–Munk converted diffuse reflectance spectrum of crystal **1a**.

7. NMR Charts

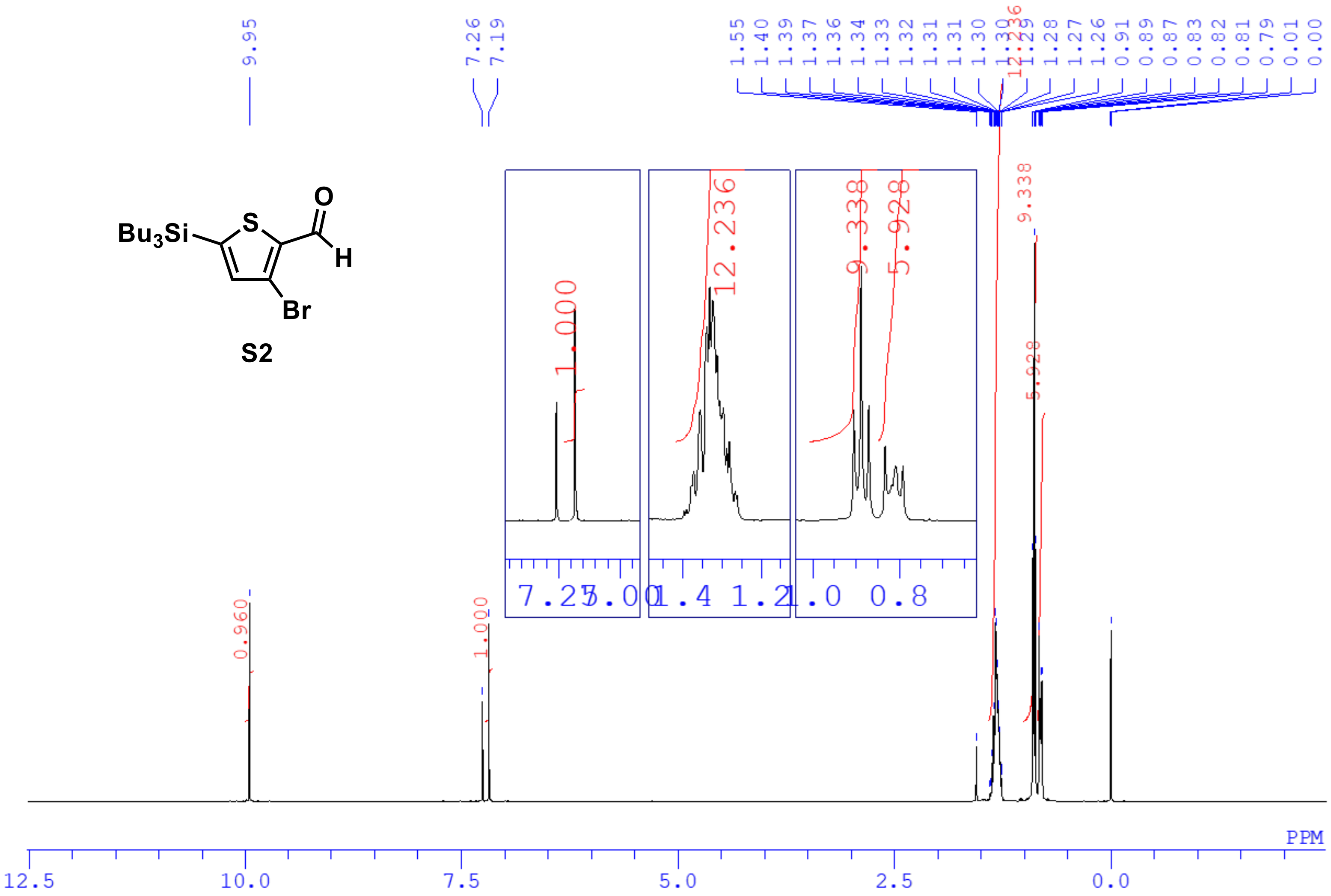

S2

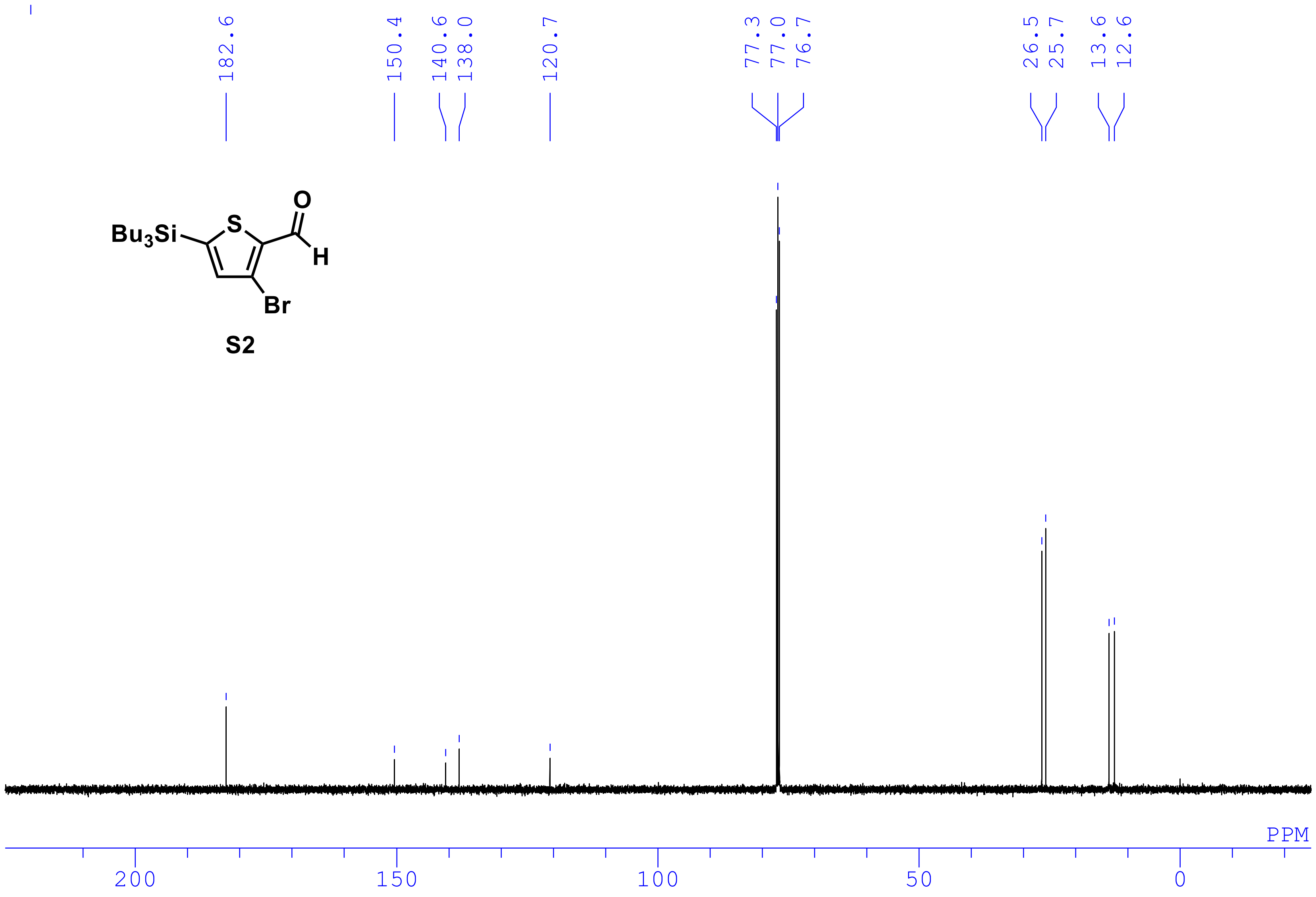

182.6
150.4
140.6
138.0
120.7
77.3
77.0
76.7
26.5
25.7
13.6
12.6
Bu3Si
S
O
H
Br
S2
PPM
200
150
100
50
0

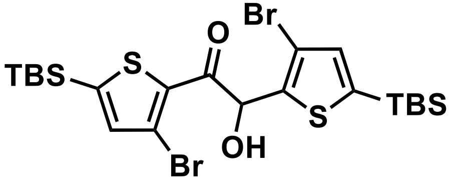
TBS
S
O
Br
S
TBS
Br
OH

S4

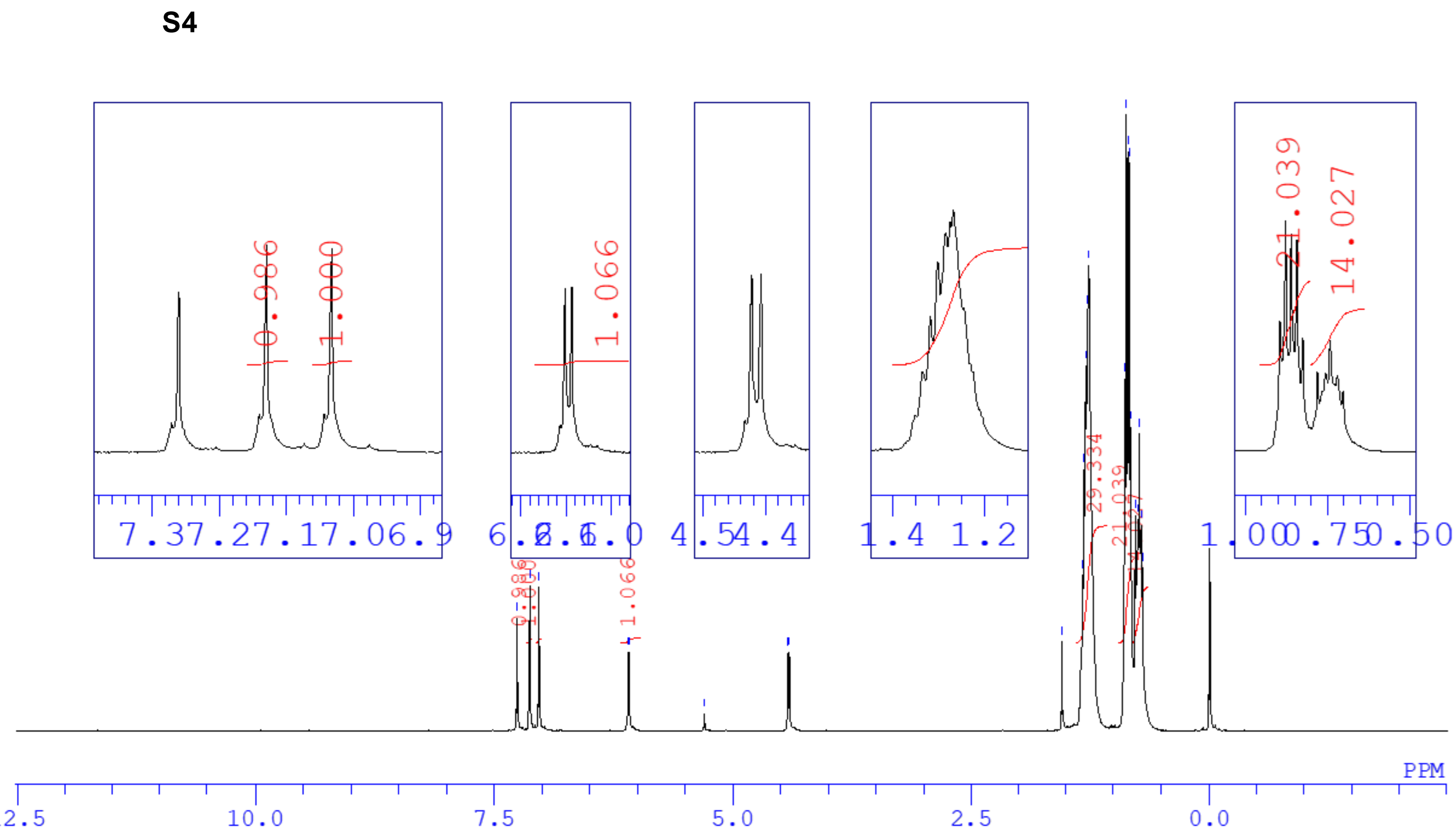
7.26
7.13
7.03
6.10
6.09
5.30
4.42
4.41
1.55
1.34
1.32
1.30
1.29
1.28
1.27
0.90
0.88
0.86
0.84
0.83
0.78
0.76
0.74
0.72
0.70
0.00
0.986
1.000
1.066
21.039
14.027
29.334
PPM
12.5
10.0
7.5
5.0
2.5
0.0

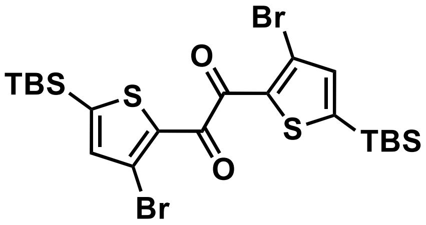
TBS
S
Br
O
O
S
TBS
Br

1c

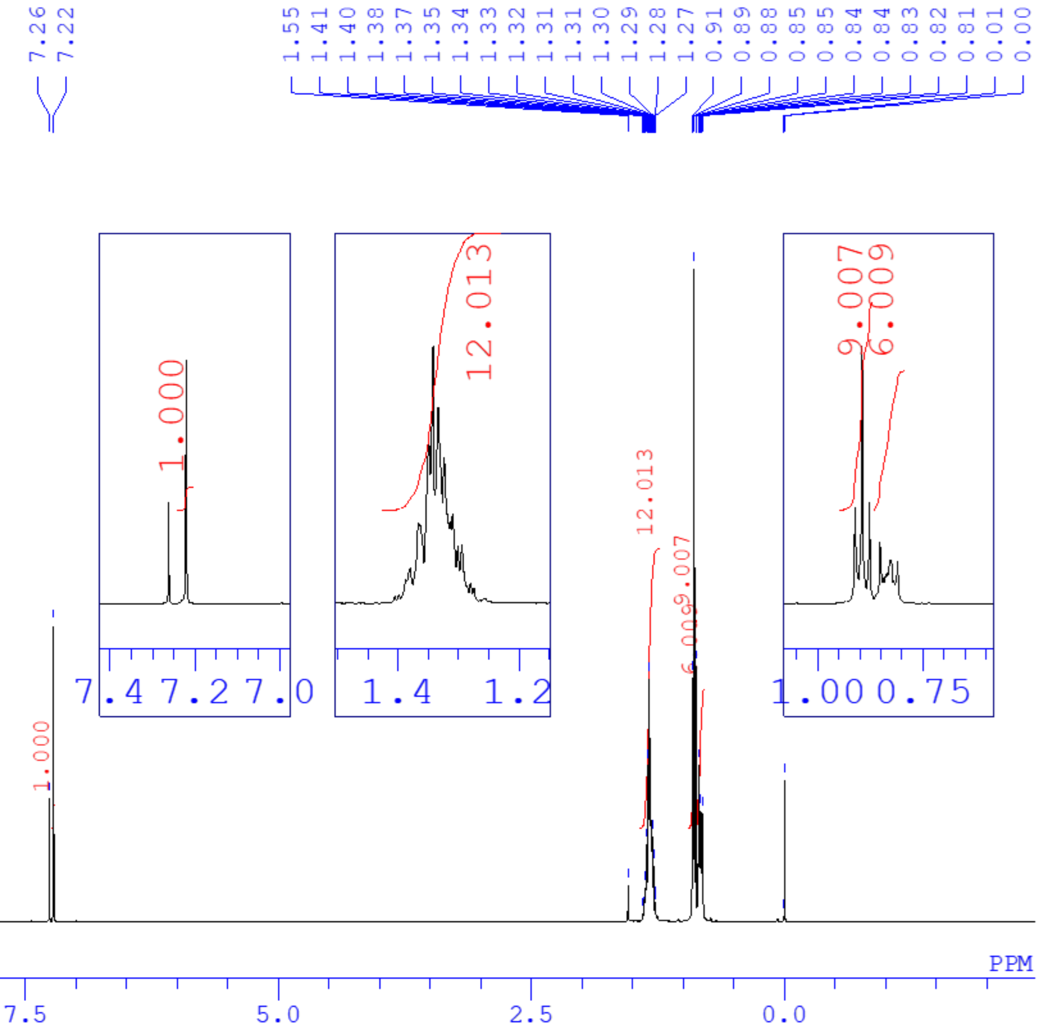
7.26
7.22
1.55
1.41
1.40
1.38
1.37
1.35
1.34
1.33
1.32
1.31
1.31
1.30
1.29
1.28
1.27
0.91
0.89
0.88
0.85
0.85
0.84
0.84
0.83
0.82
0.81
0.01
0.00
1.000
12.013
9.007
6.009
PPM
12.5
10.0
7.5
5.0
2.5
0.0

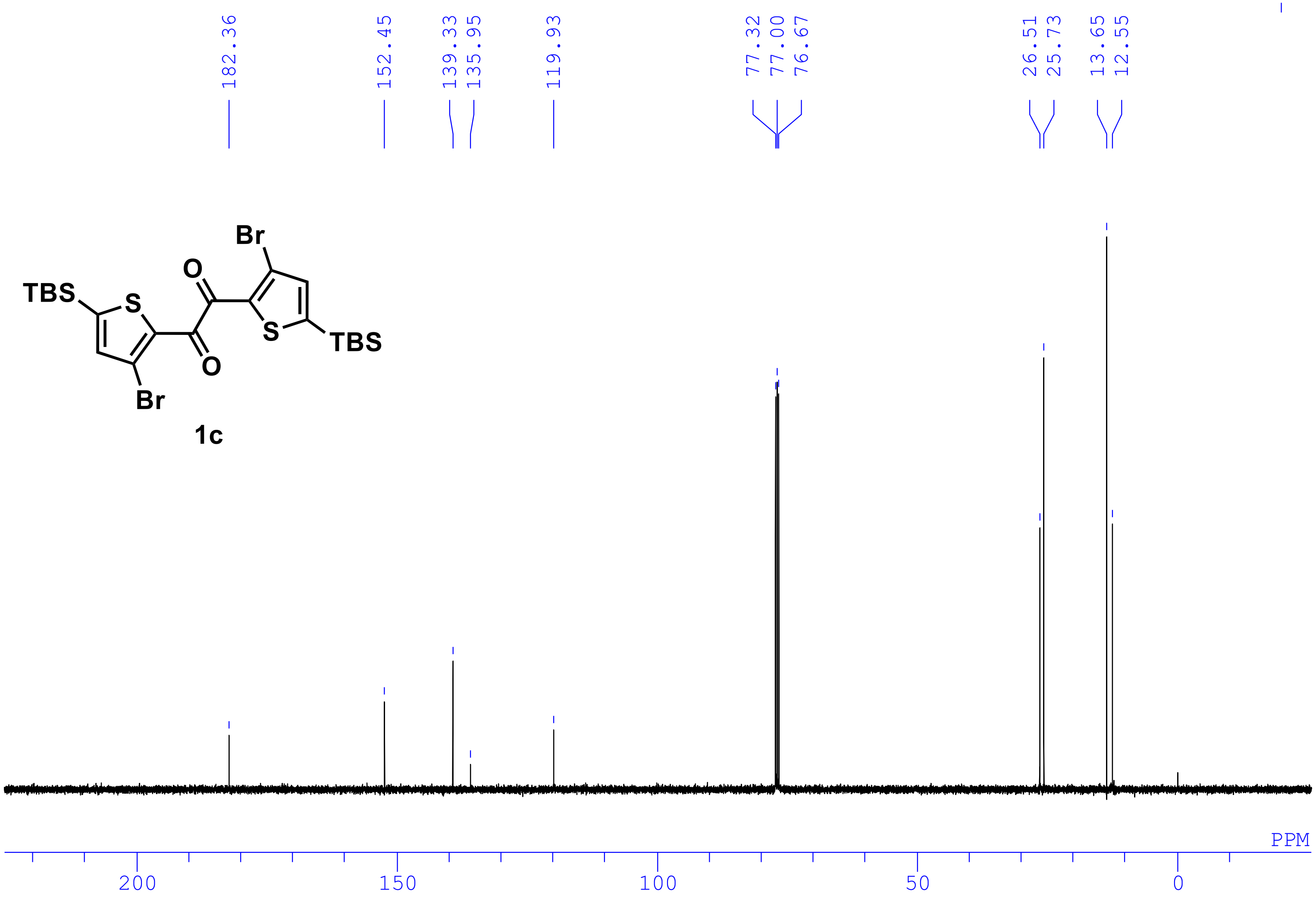
182.36
152.45
139.33
135.95
119.93
77.32
77.00
76.67
26.51
25.73
13.65
12.55
PPM
200
150
100
50
0
Br
O
TBS
S
S
TBS
O
Br

1c

8. References

1. G. Favaro, F. Masetti and A. Romani, *J. Photochem. Photobiol. A*, 1990, **53**, 41-49.
2. L. Flamigni, F. Barigelletti, S. Dellonte and G. Orlandi, *J. Photochem.*, 1983, **21**, 237-244.
3. Y. Tsuboi, K. Okada, S. Ishizaka and N. Kitamura, *Anal. Sci.*, 2005, **21**, 303-308.
4. J. Xu, A. Takai, Y. Kobayashi and M. Takeuchi, *Chem. Commun.*, 2013, **49**, 8447-8449.
5. B. Ventura, A. Bertocco, D. Braga, L. Catalano, S. d'Agostino, F. Grepioni and P. Taddei, *J. Phys. Chem. C*, 2014, **118**, 18646-18658.
6. J. J. Donkerbroek, J. J. Elzas, C. Gooijer, R. W. Frei and N. H. Velthorst, *Talanta*, 1981, **28**, 717-723.
7. M. Szymanski, R. P. Steer and A. Maciejewski, *Chem. Phys. Lett.*, 1987, **135**, 243-248.
8. M. Szymanski, A. Maciejewski and R. P. Steer, *J. Phys. Chem.*, 1988, **92**, 2485-2489.
9. G. D. Gutierrez, G. T. Sazama, T. Wu, M. A. Baldo and T. M. Swager, *J. Org. Chem.*, 2016, **81**, 4789-4796.
10. Z. Yu, Y. Wu, L. Xiao, J. Chen, Q. Liao, J. Yao and H. Fu, *J. Am. Chem. Soc.*, 2017, **139**, 6376-6381.
11. A. B. Pangborn, M. A. Giardello, R. H. Grubbs, R. K. Rosen and F. J. Timmers, *Organometallics*, 1996, **15**, 1518-1520.
12. Y. Tani, M. Terasaki, M. Komura and T. Ogawa, *J. Mater. Chem. C*, 2019, **7**, 11926-11931.
13. (a) K. Suzuki, A. Kobayashi, S. Kaneko, K. Takehira, T. Yoshihara, H. Ishida, Y. Shiina, S. Oishi and S. Tobita, *Phys. Chem. Chem. Phys.*, 2009, **11**, 9850–9860; (b) A. M. Brouwer, *Pure Appl. Chem.*, 2011, **83**, 2213-2228.
14. S. Miyazaki, K. Miyata, H. Sakamoto, F. Suzue, Y. Kitagawa, Y. Hasegawa and K. Onda, *J. Phys. Chem. A*, 2020, **124**, 6601-6606.
15. M. Saigo, Y. Shimoda, T. Ehara, T. Ryu, K. Miyata and K. Onda, *Bull. Chem. Soc. Jpn.*, 2022, **95**, 381-388.
16. (a) G. H. Allen, R. P. White, D. P. Rillema and T. J. Meyer, *J. Am. Chem. Soc.*, 1984, **106**, 2613-2620; (b) J. V. Caspar, T. D. Westmoreland, G. H. Allen, P. G. Bradley, T. J. Meyer and W. H. Woodruff, *J. Am. Chem. Soc.*, 1984, **106**, 3492-3500; (c) J. Yanagisawa, T. Hiraoka, F. Kobayashi, D. Saito, M. Yoshida, M. Kato, F. Takeiri, G. Kobayashi, M. Ohba, L. F. Lindoy, R. Ohtani and S. Hayami, *Chem.*

Commun., 2020, **56**, 7957-7960.

17. K. Huang, A. Rhys and N. F. Mott, *Proc. R. Soc. Lond. A*, 1950, **204**, 406-423.
18. (a) C. A. Parker and W. T. Rees, *Analyst*, 1960, **85**, 587-600; (b) J. Mooney and P. Kambhampati, *J. Phys. Chem. Lett.*, 2013, **4**, 3316-3318.
19. (a) M. J. Frisch, G. W. Trucks, H. B. Schlegel, G. E. Scuseria, M. A. Robb, J. R. Cheeseman, G. Scalmani, V. Barone, G. A. Petersson, H. Nakatsuji, X. Li, M. Caricato, A. V. Marenich, J. Bloino, B. G. Janesko, R. Gomperts, B. Mennucci, H. P. Hratchian, J. V. Ortiz, A. F. Izmaylov, J. L. Sonnenberg, D. Williams-Young, F. Ding, F. Lipparini, F. Egidi, J. Goings, B. Peng, A. Petrone, T. Henderson, D. Ranasinghe, V. G. Zakrzewski, J. Gao, N. Rega, G. Zheng, W. Liang, M. Hada, M. Ehara, K. Toyota, R. Fukuda, J. Hasegawa, M. Ishida, T. Nakajima, Y. Honda, O. Kitao, H. Nakai, T. Vreven, K. Throssell, J. A. Montgomery Jr., J. E. Peralta, F. Ogliaro, M. J. Bearpark, J. J. Heyd, E. N. Brothers, K. N. Kudin, V. N. Staroverov, T. A. Keith, R. Kobayashi, J. Normand, K. Raghavachari, A. P. Rendell, J. C. Burant, S. S. Iyengar, J. Tomasi, M. Cossi, J. M. Millam, M. Klene, C. Adamo, R. Cammi, J. W. Ochterski, R. L. Martin, K. Morokuma, O. Farkas, J. B. Foresman and D. J. Fox, *Gaussian 16 Rev. C.01*, Wallingford, CT, 2019; (b) S. Grimme, J. Antony, S. Ehrlich and H. Krieg, *J. Chem. Phys.*, 2010, **132**.
20. (a) F. Neese, *Wiley Interdiscip. Rev.: Compt. Mol. Sci.*, 2012, **2**, 73-78; (b) F. Neese, *Wiley Interdiscip. Rev.: Compt. Mol. Sci.*, 2018, **8**, e1327; (c) S. Grimme, S. Ehrlich and L. Goerigk, *J. Comput. Chem.*, 2011, **32**, 1456-1465.
21. M. J. G. Peach and D. J. Tozer, *J. Phys. Chem. A*, 2012, **116**, 9783-9789.
22. S. Alvarez, *Dalton Transactions*, 2013, **42**, 8617-8636.
